# Cultivar Diversity of Grape Skin Polyphenol Composition and Changes in Response to Drought Investigated by LC-MS Based Metabolomics

**DOI:** 10.3389/fpls.2017.01826

**Published:** 2017-10-27

**Authors:** Lucie Pinasseau, Anna Vallverdú-Queralt, Arnaud Verbaere, Maryline Roques, Emmanuelle Meudec, Loïc Le Cunff, Jean-Pierre Péros, Agnès Ageorges, Nicolas Sommerer, Jean-Claude Boulet, Nancy Terrier, Véronique Cheynier

**Affiliations:** ^1^Plateforme Polyphénols SPO, INRA, Montpellier SupAgro, Université de Montpellier, Montpellier, France; ^2^SPO, INRA, Montpellier SupAgro, Université de Montpellier, Montpellier, France; ^3^IFV Pôle national matériel végétal, UMT Génovigne, Montpellier, France; ^4^AGAP, INRA, CIRAD, Montpellier SupAgro, Université de Montpellier, Montpellier, France

**Keywords:** grape berry, *Vitis vinifera*, phenolic compounds, UHPLC-QqQ-MS, metabolomics, water deficit, large-scale studies

## Abstract

Phenolic compounds represent a large family of plant secondary metabolites, essential for the quality of grape and wine and playing a major role in plant defense against biotic and abiotic stresses. Phenolic composition is genetically driven and greatly affected by environmental factors, including water stress. A major challenge for breeding of grapevine cultivars adapted to climate change and with high potential for wine-making is to dissect the complex plant metabolic response involved in adaptation mechanisms. A targeted metabolomics approach based on ultra high-performance liquid chromatography coupled to triple quadrupole mass spectrometry (UHPLC-QqQ-MS) analysis in the Multiple Reaction Monitoring (MRM) mode has been developed for high throughput profiling of the phenolic composition of grape skins. This method enables rapid, selective, and sensitive quantification of 96 phenolic compounds (anthocyanins, phenolic acids, stilbenoids, flavonols, dihydroflavonols, flavan-3-ol monomers, and oligomers…), and of the constitutive units of proanthocyanidins (i.e., condensed tannins), giving access to detailed polyphenol composition. It was applied on the skins of mature grape berries from a core-collection of 279 *Vitis vinifera* cultivars grown with or without watering to assess the genetic variation for polyphenol composition and its modulation by irrigation, in two successive vintages (2014–2015). Distribution of berry weights and δ^13^C values showed that non irrigated vines were subjected to a marked water stress in 2014 and to a very limited one in 2015. Metabolomics analysis of the polyphenol composition and chemometrics analysis of this data demonstrated an influence of water stress on the biosynthesis of different polyphenol classes and cultivar differences in metabolic response to water deficit. Correlation networks gave insight on the relationships between the different polyphenol metabolites and related biosynthetic pathways. They also established patterns of polyphenol response to drought, with different molecular families affected either positively or negatively in the different cultivars, with potential impact on grape and wine quality.

## Introduction

In the context of climate change, it is of prime importance to anticipate and predict the response of the different biota to the changes in environmental conditions, especially for plants, that are devoid of motility. Climate change is expected to affect plant composition and consequently, in the case of crop species such as grapevine, the quality of plant derived products. Among plant metabolites, secondary metabolites, including phenolic compounds, have been recognized as playing multiple roles in plant response to a wide range of biotic and abiotic stresses and in particular to water stress (Baker and Orlandi, 1995; Dixon and Paiva, 1995; Caldwell et al., 2003). They are also essential components of plant derived foods and beverages, responsible for major organoleptic properties such as color and taste and contributing health benefit (Manach et al., 2004).

Grape phenolic compounds comprise several families, divided between non flavonoids (hydroxybenzoic acids, hydroxycinnamic acids, and stilbenes) and flavonoids, based on the same C6-C3-C6 skeleton (flavonols, dihydroflavonols, flavan-3-ols, and anthocyanins). Each family is represented by several compounds differing by their hydroxylation level and by substitution of the hydroxy groups (methylation, glycosylation, acylation). For example, anthocyanins, the red grape pigments, are based on six aglycones which can be mono- or di-glucosylated and further acylated with acetic, *p*-coumaric, and caffeic acid, giving rise to a large number of compounds (Favretto and Flamini, 2000; Heier et al., 2002; Vidal et al., 2004a). Moreover, various anthocyanin derivatives such as anthocyanin dimers and flavan-3-ol anthocyanin adducts have been detected in grape skin extracts (Vidal et al., 2004b). Grape flavan-3-ols also show high diversity. They include several monomers (catechin, epicatechin, gallocatechin, epigallocatechin, and epicatechin 3-gallate) that are the constitutive units of oligomers and polymers (proanthocyanidins or condensed tannins), with degrees of polymerization ranging from 2 to over 100 in grape skin (Souquet et al., 1996).

The impact of water stress on grape berry composition has already been investigated (reviewed in Downey et al., 2006; Teixeira et al., 2013). However, those studies were performed on a few elite cultivars (e.g., Cabernet-Sauvignon, Chardonnay, Syrah, Merlot…) analyzed for a limited number of phenolic metabolites, most often anthocyanins. In addition, results are hardly comparable between studies since differences in water regime were not applied at the same developmental stage and with the same intensity, and amounts of phenolic compounds were not expressed in the same units. Since water stress induces a decrease of berry size, and given that most of phenolic compounds are stored in external cell layers of the cells, an increase of phenolic concentration expressed as mg/g of fresh weight can be measured without any increase of content expressed in mg/berry (Bucchetti et al., 2011). As a general trend, water stress was shown to induce an increase of anthocyanin content and a qualitative modification of the anthocyanin pool, when fine analysis was performed (Castellarin et al., 2007; Bucchetti et al., 2011; Ollé et al., 2011; Hochberg et al., 2015). In contrast, conflicting results were obtained on other classes of phenolic compounds. For example, no (Kennedy et al., 2002; Ollé et al., 2011) or slight (Ojeda et al., 2002) modifications in flavan-3-ol composition and a reduction (Hochberg et al., 2015; Savoi et al., 2017) or increase (Deluc et al., 2011; Herrera et al., 2017) of stilbene accumulation have been observed in response to water deficit. Cultivar specificity of these responses has been reported by comparing cv. Chardonnay (Deluc et al., 2009) or cv. Syrah (Hochberg et al., 2015) to cv. Cabernet Sauvignon. This may be related to hydraulic behavior or to differences in phenological stages (Hochberg et al., 2015) as early and late water deficit affect phenolic composition in different ways (Ojeda et al., 2002; Ollé et al., 2011; Casassa et al., 2015).

Nevertheless, a major challenge for breeding of grapevine cultivars adapted to climate change and with high potential for wine-making is to describe and dissect the complex global phenolic response involved in adaptation mechanisms on a wide range of genotypes. The aim of the present study was to investigate the polyphenol composition and its modification in response to water deficit on a large panel of cultivars reflecting the genetic diversity of grapevines.

## Materials and methods

### Plant material and experimental design

The diversity panel (DP) of 279 *V. vinifera* cultivars described by Nicolas et al. (2016) was used for this study. It is composed of three subgroups of 93 cultivars representing the three main genetic pools, which differ in use and geographical origin: wine West (WW), wine East (WE), table East (TE).

Each cultivar was over-grafted in 2009 on 6-years old vines of cultivar Marselan in a complete randomized block design with five blocks and one plant of each cultivar per block. The trial was located at the Domaine du Chapitre of Montpellier Supagro (Villeneuve-les-Maguelonne, France), maintained under classical local training system (double cordon, 4,000 plants/ha). A drip irrigation was installed in two blocks in order to create a water contrast with the other three blocks. In 2014 and 2015, irrigation was applied 2 days per week from the last third of June to the end of the berry sampling period (October 6th and October 16th, in 2014 and 2015, respectively). The quantity of supplied water was approximately of 10 mm per 10-day period. Data on total rainfall per 10-day period were obtained for the nearest climatic station.

### Sampling

Grape berries were collected at ripeness when sugar concentration reached 20°Brix. To determine this sampling stage, regular measurements (three times a week from week 30) were performed with an optical refractometer using a few berries per cultivar/treatment. Three clusters were sampled per cultivar/treatment, their end parts were discarded and 100 berries randomly sampled to estimate mean berry weight. Thirty berries were then randomly selected and their skins isolated, frozen in liquid nitrogen, and stored at −80°C until extraction and analysis. The remaining berries were crushed and the juice was filtered. An aliquot of 1 mL was prepared for the analysis of the 13C/12C ratio (δ*13*C).

### δ^13^C analysis

δ^13^C or carbon isotope discrimination is expressed compared to a standard and ranges at maturity stage from −27 p. 1000 (no water deficit) to −20 p. 1000 (severe water deficit stress, Van Leeuwen et al., 2001). Its measurement was subcontracted. Samples were freeze-dried, pre-weighed, encapsulated, and then sent to OEA Laboratories Limited (Cornwall, UK). They were analyzed by a Sercon 20-20 dual turbo pumped Continuous Flow Isotope Ratio Mass Spectrometer (CF/IRMS) linked to a Thermo EA1110 Elemental Analyzer (EA) NC dual tube configured fitted with a high performance Carbosieve G separation column. Samples and references were weight optimized for δ13C analysis according to elemental composition. IRMS calibration was scale normalized using isotope references USGS-40 and USGS-41a as lower and upper scale anchors with random QC sample checks within sample sequences. Absolute weights of carbon in samples were determined from the IRMS total beam values relative to the elemental composition of the references. References were weighed from bulk material to 6 decimal places using a Mettler UMX5 microbalance. Standard deviations for isotope reference materials was typically better than 0.15 for carbon.

### Extraction and sample preparation for polyphenol analysis

#### Extraction

The extraction procedure was adapted from that of Mané et al. (2007), as described by Pinasseau et al. (2016). Briefly, frozen skins were ground with liquid nitrogen with a Mortar Grinder Pulverisette 2 (Fritsch, Idar-Oberstein, Germany). One hundred milliliters of of powder were weighed and 500 μL of methanol was immediately added. Then 3.5 mL of acetone/H2O 70/30 (v/v) 0.05% trifluoroacetic acid were added. The mixture was crushed with Precellys (Bertin Technologies, Montigny-le-Bretonneux, France) during three cycles (3 × 40 s each). 3.5 mL were centrifuged with a Heraeus Multifuge X3R Centrifuge (ThermoFischer Scientific, Waltham, USA) (21,320 g, 5 min, 4°C). Aliquots (1 mL) of the supernatant were dried with Genevac (SP Scientific, Warminster, PA, USA).

#### Sample preparation for determination of polyphenol composition

Five hundred microliters of methanol/H_2_O 50/50 (v/v) 1% formic acid were added on the solid obtained after evaporation with Genevac (SP Scientific, Warminster, PA, USA). After solubilization using an Ultrasonic Cleaner (VWR, Fontenay-sous-Bois, France) (30 min), the solution obtained was centrifuged with Hettich Mikro 220R (Hettich Lab Technology, Tuttlingen, Germany) (15,000 rpm, 15 min, 4°C). Dilutions 1/20 were prepared. Pure and diluted samples were injected in triplicate for UHPLC-QqQ-MS analysis.

The phloroglucinolysis reaction was carried out on the solid obtained after evaporation with Genevac (SP Scientific, Warminster, PA, USA), following the procedure described in Pinasseau et al. (2016).

### Instrumentation

Analyses were carried out using an Acquity UPLC system (Waters, Saint-Quentin-en-Yvelines, France) hyphenated to a triple quadrupole (QqQ) TQD mass spectrometer (Waters, Saint-Quentin-en-Yvelines, France). The UPLC system included a binary pump, a cooled autosampler maintained at 7°C and equipped with a 5-μL sample loop, a 100-μL syringe and a 30-μL needle, and a diode array detection (DAD). The DAD spectra were recorded in the range of 210–600 nm (resolution 1.2 nm). MassLynx software was used to control the instruments and to acquire the data which were then processed with the TargetLynx software.

#### Chromatographic conditions

The column used for chromatographic separation was a reversed-phase Acquity HSS T3 1.8 μm 1.0 × 100 mm (Waters, Saint-Quentin-en-Yvelines, France) protected by a 0.2 μm in-line filter and maintained at 40°C. The mobile phase consisted of 1% (v/v) formic acid in deionized water (solvent A) and 1% (v/v) formic acid in methanol (solvent B). The flow rate was 0.170 mL/min. Samples were injected into the column by using the Partial Loop with Needle Overfill injection mode with an injection volume of 1 μL.

##### UPLC analysis of polyphenol composition

Isocratic 1%B from 0.0 to 2.0 min, linear 1–5%B from 2.0 to 2.1 min, linear 5–10%B from 2.1 to 8.0 min, linear 10–28%B from 8.0 to 12.0 min, isocratic 28%B from 12.0 to 18.0 min, linear 28–45%B from 18.0 to 22.0 min, linear 45–99%B from 22.0 to 23.5 min, isocratic 99%B from 23.5 to 26.5 min. At the end of this sequence, the column was brought back to initial conditions with linear 99–1%B from 26.5 to 27.0 min, then re-equilibrated with isocratic 1%B from 27.0 to 30.0 min.

##### UPLC analysis of tannin units after phloroglucinolysis

Isocratic 2%B from 0.0 to 1.5 min, linear 2–7%B from 1.5 to 3.0 min, linear 7–40%B from 3.0 to 5.0 min, linear 40–99%B from 5.0 to 6.0 min, isocratic 99%B from 6.0 to 6.5 min. As the end of this sequence, the column was brought back to initial conditions with linear 99–2%B from 6.5 to 7.0 min, then re-equilibrated with isocratic 1%B from 7.0 to 10.0 min.

#### Mass spectrometry conditions

The mass spectrometer was operated in MRM mode with electrospray ionization (ESI) either in positive or negative ionization mode. The source and desolvation temperatures were respectively set at 120 and 450°C. Nitrogen was used as desolvation (500 L/h) and cone (50 L/h) gas. Argon was used as collision gas at a flow rate of 0.16 mL/min. Capillary voltage was set at 3.5 kV in positive mode and 2.8 kV in negative mode.

### Polyphenol composition data

Lower molecular weight phenolic compounds including phenolic acids, stilbenes, anthocyanins, flavonols, dihydroflavonols, flavan3-ol monomers, dimers and trimers, and derived pigments and tannins, were analyzed by UHPLC-QqQ-MS in the MRM mode, using a method adapted from that described by Lambert et al. (2015). A few additional phenolic compounds detected in the grape extracts were identified and included in the method as detailed below. Glutathione in its reduced and oxidized forms was analyzed by UHPLC-QqQ-MS in the MRM mode as described by Vallverdú-Queralt et al. (2015). Flavan-3-ol units released after phloroglucinolysis were analyzed by UHPLC-QqQ-MS in the MRM mode (Lambert et al., 2015).

MRM transitions parameters of added target compounds that are commercially available were optimized by using the Intellistart tool of the Masslynx software which consists in automatically detecting the major fragments and optimizing cone voltages and collision energies. 1-galloyl-β-D-glucose (glucogallin) was characterized by the loss of glucose (−162Th). The main fragment (*m/z* 139Th) of (-)-epigallocatechin was the result of a Retro-Diels-Alder (RDA) fragmentation. Piceatannol was characterized by the loss of a diphenol (−110Th). These three molecules and quercetin-3-*O*-glucuronide were included in the calibration standards.

For new target analytes that are not commercially available, MRM parameters were optimized directly in grape extracts and compared to data reported in the literature. Pelargonidin 3-glucoside was characterized by the loss of glucose (−162Th) (Arapitsas et al., 2012) while pelargonidin 3-acetylglucoside and pelargonidin 3-coumaroylglucoside were characterized by the loss of the acetylglucose (−204Th) and coumaroylglucose (−308Th), respectively. These fragmentation patterns were specifically targeted in accordance with those of the other anthocyanins described by Lambert et al. (2015). Transitions of (epi)gallocatechin-malvidin 3-glucoside and (epi)gallocatechin-peonidin 3-glucoside were specifically targeted in accordance with their non galloylated equivalents described in Lambert et al. (2015). They are characterized by the loss of glucose (−162Th). Analysis of anthocyanin-tannin (A-T) bicyclic A-type adducts, namely peonidin 3-glucoside-(epi)catechin (*m/z* 753Th), petunidin 3-glucoside-(epi)catechin (*m/z* 769Th), malvidin 3-glucoside-(epi) catechin (*m/z* 783Th), malvidin 3-glucoside-(epi)gallocatechin (*m/z* 799Th), was optimized in the same way. The main fragments detected at *m/z* 313, 329, and 343Th, respectively for peonidin, petunidin, and malvidin derived A-T adducts result from a retro Diels-Alder (RDA) fragmentation (−168Th), the loss of the anthocyanin A-ring (−126Th) and that of the glucose substituent (−162Th) (Remy-Tanneau et al., 2003). Malvidin 3-glucoside dimer and malvidin 3-glucoside-peonidin 3-glucoside were characterized by the loss of the two glucose moieties (−324Th) (Vidal et al., 2004b). Glucosylated flavonols such as isorhamnetin 3-glucoside, kaempferol 3-glucoside, and syringetin 3-glucoside were qualified by the loss of the glucose (−162Th) (Vrhovsek et al., 2012). Fragmentation of laricitrin 3-glucoside (−162Th) was specifically targeted in accordance with fragmentation patterns of the other glucosylated flavonols (Lambert et al., 2015). Kaempferol 3-glucuronide was qualified by the loss of the glucuronide (−176Th) (Vrhovsek et al., 2012). Fragmentations of isorhamnetin 3-glucuronide, laricitrin 3-glucuronide, and syringetin 3-glucuronide (loss of the glucuronide −176Th) were optimized in accordance with the fragmentation pattern of the other glucuronidated flavonols (Lambert et al., 2015). Piceatannol 3-glucoside was characterized by the loss of the glucose (−162Th) (Vrhovsek et al., 2012). Fragmentation of (+)-gallocatechin (fragment at *m/z* 139Th after a RDA fragmentation) was optimized in accordance with (–)-epigallocatechin which is commercially available. Anthocyanins were expressed as equivalent malvidin 3-*O*-glucoside. Flavonol glucosides and flavonol glucuronides were expressed as equivalent quercetin 3-glucoside and quercetin 3-glucuronide, respectively. Piceatannol glucoside was expressed as equivalent piceid.

Quantitative data on 105 compounds was thus obtained. In addition, 17 variables have been calculated, including quantitative variables, namely total concentrations of native anthocyanins (s_AN_n), flavonols (s_FO), stilbenes (s_ST), hydroxycinnamic acid derivatives (s_HC), hydroxybenzoic acid derivatives (s_HB), flavan-3-ols (i.e., sum of tannin units released after phloroglucinolysis, s_FA), and qualitative variables, %acylated anthocyanins (p_AN_acyl), %B-ring trihydroxylated anthocyanins (p_AN_tri), %B-ring methylated anthocyanins (p_AN_met), %B-ring monohydroxylated flavonols (p_FO_mono), %B-ring dihydroxylated flavonols (p_FO_di), %B-ring trihydroxylated flavonols (p_FO_tri), %B-ring methylated flavonols (p_FO_met), %flavonol glucuronides (p_FO-glucur), %B-ring trihydroxylated flavan-3-ol units (p_FA_tri) %galloylated flavan-3-ol units (p_FA_gall), mean degree of polymerization (dp_FA), calculated as the molar ratio of total released units to total terminal units. The list of variables is given in Table 1, along with their codes and abbreviations.

**Table 1:** list of variables, variable codes, and abbreviations.

**Code**	**Abbreviation**	**Full name**
**AN–NATIVE ANTHOCYANINS+DIMERS:**		
1	AN-Pg-glc	pelargonidin 3-glucoside
2	AN-Cy-glc	cyanidin 3-glucoside
3	AN-Dp-glc	delphinidin 3-glucoside
4	AN-Pt-glc	petunidin 3-glucoside
5	AN-Pn-glc	peonidin 3-glucoside
6	AN-Mv-glc	malvidin3-glucoside
7	AN-Cy-diglc	cyanidin 3,5-diglucoside
8	AN-Dp-diglc	delphinidin 3,5-diglucoside
9	AN-Pt-diglc	petunidin 3,5-diglucoside
10	AN-Pn-diglc	peonidin 3,5-diglucoside
11	AN-Mv-diglc	malvidin 3,5-diglucoside
12	AN-Pg-acglc	pelargonidin 3-acetylglucoside
13	AN-Cy-acglc	cyanidin 3-acetylglucoside
14	AN-Dp-acglc	delphinidin 3-acetylglucoside
15	AN-Pt-acglc	petunidin 3-acetylglucoside
16	AN-Pn-acglc	peonidin 3-acetylglucoside
17	AN-Mv-acglc	malvidin 3-acetylglucoside
18	AN-Pg-coumglc	pelargonidin 3-*p*-coumaroylglucoside
19	AN-Cy-coumglc	cyanidin 3-*p*-coumaroylglucoside
20	AN-Dp-coumglc	delphinidin 3-*p*-coumaroylglucoside
21	AN-Pt-coumglc	petunidin 3-*p*-coumaroylglucoside
22	AN-Pn-coumglc	peonidin 3-*p*-coumaroylglucoside
23	AN-Mv-coumglc	malvidin3-*p*-coumaroylglucoside
24	AN-Cy-caffglc	cyanidin 3-caffeoylglucoside
25	AN-Dp-caffglc	delphinidin 3-caffeoylglucoside
26	AN-Pt-caffglc	petunidin 3-caffeoylglucoside
27	AN-Pn-caffglc	peonidin 3-caffeoylglucoside
28	AN-Mv-caffglc	malvidin 3-caffeoylglucoside
29	AN-Mv-glc-Pn-glc	malvidin 3-glucoside-peonidin 3-glucoside
30	AN-Mv-glc-dimer	malvidin 3-glucoside dimer
**AP–PYRANO ANTHOCYANINS**		
31	AP-py-Pn-glc	pyranopeonidine 3- glucoside
32	AP-py-Mv-glc	pyranomalvidin 3-glucoside (vitisin B)
33	AP-hp-py-Pn-glc	*p*-hydroxyphenylpyranopeonidin3-glucoside
34	AP-hp-py-Mv-glc	*p*-hydroxyphenylpyranomalvidin 3-glucoside
35	AP-ctc-py-Pn-glc	catechylpyranopeonidin 3-glucoside
36	AP-ctc-py-Mv-glc	catechylpyranomalvidin 3-glucoside (pinotin A)
37	AP-cbx-py-Pn-glc	carboxypyranopeonidin 3-glucoside
38	AP-cbx-py-Mv-glc	carboxypyranomalvidin 3-glucoside (vitisin A)
**AF–ANTHOCYANINS-FLAVANOLS**:		
39	AF-Pt-glc-(epi)cat	petunidin 3-glucoside-(epi)catechin A-F bicyclic
40	AF-Pn-glc-(epi)cat	peonidin 3-glucoside-(epi)catechin A-F bicyclic
41	AF-Mv-glc-(epi)gallocat	malvidin 3-glucoside-(epi)gallocatechin A-F bicyclic
42	AF-Mv-glc-(epi)cat	malvidin 3-glucoside-(epi)catechin A-F bicyclic
43	AF-(epi)gallocat-Pn-glc	(epi)gallocatechin-peonidin 3-glucoside F-A (2 isomers)
44	AF-(epi)gallocat-Mv-glc	(epi)gallocatechin-malvidin 3-glucoside F-A (2 isomers)
45	AF-(epi)cat-Pn-glc	(epi)catechin-peonidin 3-glucoside F-A (2 isomers)
46	AF-(epi)cat-Mv-glc	(epi)catechin-malvidin 3-glucoside F-A (2 isomers)
47	AF-(epi)cat-eth-Pn-glc-i1	(epi)catechin-ethyl-peonidin 3-glucoside (isomer 1)
48	AF-(epi)cat-eth-Pn-glc-i2	(epi)catechin-ethyl-peonidin 3-glucoside (isomer 2)
49	AF-(epi)cat-eth-Pn-glc-i3	(epi)catechin-ethyl-peonidin 3-glucoside (isomer 3)
50	AF-(epi)cat-eth-Pn-glc-i4	(epi)catechin-ethyl-peonidin 3-glucoside (isomer 4)
51	AF-(epi)cat-eth-Mv-glc-i1	(epi)catechin-ethyl-malvidin 3-glucoside (isomer 1)
52	AF-(epi)cat-eth-Mv-glc-i2	(epi)catechin-ethyl-malvidin 3-glucoside (isomer 2)
53	AF-(epi)cat-eth-Mv-glc-i3+4	(epi)catechin-ethyl-malvidin 3-glucoside (isomers 3 & 4)
**AC–CAFTARIC-ANTHOCYANINS:**		
54	AC-caft-Pn-glc	caftaric-peonidin 3-glucoside (2 isomers)
55	AC-caft-Mv-glc	caftaric-malvidin 3-glucoside (2 isomers)
**HF–DIHYDROFLAVONOLS**		
56	HF-taxif	taxifolin
57	HF-astilb	astilbin
**FO–FLAVONOLS**		
58	FO-syring-glucur	syringetin 3-glucuronide
59	FO-syring-glc	syringetin 3-glucoside
60	FO-querc-glucur	quercetin 3-glucuronide
61	FO-querc-glc	quercetin 3-glucoside
62	FO-myric-glucur	myricetin 3-glucuronide
63	FO-myric-glc	myricetin 3-glucoside
64	FO-laric-glucur	laricitrin 3-glucuronide
65	FO-laric-glc	laricitrin 3-glucoside
66	FO-kaempf-glucur	kaempferol 3-glucuronide
67	FO-kaempf-glc	kaempferol 3-glucoside
68	FO-isorham-glucur	isorhamnetin 3-glucuronide
69	FO-isorham-glc	isorhamnetin 3-glucoside
**ST–STILBENES**		
70	ST-c-resver	*cis*-resveratrol
71	ST-t-resver	*trans*-resveratrol
72	ST-c-piceid	*cis*-piceide
73	ST-t-piceid	*trans*-piceide
74	ST-piceat-glc	piceatannol glucoside
75	ST-piceat	piceatannol
76	ST-resver-dimer	resveratrol dimers type εviniferin (2 isomers)
**FA–FLAVANOLS (TANNINS)**		
77	FA-gallocat	gallocatechin
78	FA-epigallocat	epigallocatechin
79	FA-epicat	epicatechin
80	FA-cat	catechin
81	FA-(epi)cat-eth-(epi)cat-i1	(epi)catechin-ethyl-(epi)catechin (1 isomer)
82	FA-(epi)cat-eth-(epi)cat-i2+3	(epi)catechin-ethyl-(epi)catechin (2 isomers)
83	FA-gallocat-term	gallocatechin terminal unit
84	FA-epigallocat-term	epigallocatechin terminal unit
85	FA-epicat-term	epicatechin terminal unit
86	FA-epicat-gall-term	epicatechin 3-gallate terminal unit
87	FA-cat-term	catechin terminal unit
88	FA-(epi)gallocat-phlo	epigallocatechin phloroglucinol adduct (upper unit)
89	FA-epicat-phlo	epicatechin phloroglucinol adduct (upper unit)
90	FA-epicat-gall-phlo	epicatechin 3-gallate phloroglucinol adduct (upper unit)
91	FA-cat-phlo	catechin phloroglucinol adduct (upper unit)
**HB–HYDROXYBENZOIC ACIDS**		
92	HB-glucogall	glucogallin
93	HB-vanill-ac	vanillic acid
94	HB-syring-ac	syringic acid
95	HB-protocat-ac	protocatechuic acid
96	HB-gall-ac	gallic acid
**HC–HYDROXYCINNAMIC ACIDS**		
97	HC-ct-coutar-ac	coutaric acid (*cis* & *trans* isomers)
98	HC-ct-caftar-ac	caftaric acid (*cis* & *trans* isomers)
99	HC-t-fertar-ac	*trans*-fertaric acid
100	HC-t-caffeic-ac	*trans*-caffeic acid
101	HC-t-coumar-ac	*trans*-*p*-coumaric acid
102	HC-t-ferul-ac	*trans*-ferulic acid
**OT–OTHERS**		
103	OT-OH-tyrosol	hydroxytyrosol
104	OT-GSSG	oxidized glutathione (GSSG)
105	OT-GSH	glutathione (GSH)
**PARAMETERS RELATED TO THE VINEYARD**		
	δ^13^C	Water stress
	brix	Refractive index
	Berry weight	berry weight (g)
**CALCULATED VARIABLES**		
code	formula	Full name
s_AN_n	∑AN1-AN28	total concentration of native anthocyanins
s_FO	∑FO = ∑FO1-FO12	total concentration of flavonols
s_ST	∑ST1-ST7	total concentration of stilbenes
s_HB	∑HB1-HB5	total concentration of hydroxybenzoic acids
s_HC	∑HC1-HC6	total concentration of hydroxycinnamic acids
s_TN	∑FA7-FA15	total concentration of tannin units (phloroglucinolysis)
p_AN_acyl	∑AN12-AN28/∑AN1-AN28	%acylated anthocyanins
p_AN_tri	∑Dp,Pt,Mv/∑AN1-AN28	%B-ring trihydroxylated anthocyanins
p_AN_met	∑Pt,Pn,Mv/∑AN1-AN28	%B-ring methylated anthocyanins
p_FO_mono	∑kaempf/∑FO	%B-ring monohydroxylated flavonols
p_FO_di	∑querc+isorham/∑FO	%B-ring dihydroxylated flavonols
p_FO_tri	∑myric+laric+syring/∑FO	%B-ring trihydroxylated flavonols
p_FO_met	∑isorham+laric+syring/∑FO	%B-ring methylated flavonols
p_FO-glucur	∑glucuronides/∑FO	%flavonol glucuronides
p_TN_tri	(FA7+FA8+FA12)/∑FA7-FA15	trihydroxylated flavan-3-ol units
p_TN_gall	(FA10+FA14)/∑FA7-FA15	%galloylated flavan-3-ol units
dp_ TN	∑FA7-FA15/∑FA7-FA10	mean degree of polymerization

∑Dp,Pp,Mv = AN3+AN4+AN6+AN8+AN9+AN11+AN14+AN15+AN17+AN20+AN21+AN23+AN25+AN26+AN28

∑Pt,Pn,Mv = AN4+AN5+AN6+AN9+AN10+AN11+AN15+AN16+AN17+AN21+AN22+AN23+AN26+AN27+AN28

∑kaempf = FO9+FO10

∑querc + Isorham = FO3+FO4+FO11+FO12

∑myric+laric+syring = FO1+FO2+FO5+FO6+FO7+FO8

∑isorham+laric+syring = FO1+FO2+FO7+FO8+FO11+FO12

*∑glucuronides = FO1+FO3+FO3+FO7+FO9+FO11*.

### Chemometrics

For the 2 years of sampling (2014 and 2015), chemometrics treatments were performed on the MRM data for the 105 compounds, sorted by families (same order in 2014 and 2015) anthocyanins, flavanols, stilbenes, etc. For each observation, the 105 compounds were associated to the 17 calculated parameters, and the three parameters from the vineyard: δ^13^C, refractive index, berry weight. Only cultivars for which both irrigated and non-irrigated observations were available were considered in each vintage. Samples with missing berry weight values were also eliminated. For the 105 MRM parameters, values below the quantification threshold were automatically replaced with a value corresponding to half of the threshold value.

One-way analysis of variance and principal component analysis were performed using the Fact toolbox of the Scilab software. Correlation networks were processed using Cytoscape. Hierarchical clustering of phenolic compounds and genotypes was performed using EXPANDER V6 (Sharan et al., 2003). The distance measurement used in the algorithm is (1-Pearson Correlation)/2, with complete linkage.

### Reagents and chemicals

Formic acid, HPLC grade methanol, acetone, hydrochloric acid, trifluoroacetic acid, ammonium formiate, L-ascorbic acid, and phloroglucinol were purchased from Sigma Aldrich (St Louis, MO, USA). Deionized water was obtained from a Milli-Q purification system (Millipore, Molsheim, France). Standards of (+)-catechin, (–)-epicatechin, (–)-epicatechin 3-*O*-gallate, reduced L-glutathione, oxidized L-glutathione, piceatannol, *p*-coumaric acid, protocatechuic acid, syringic acid, *trans*-caftaric acid, and *trans*-resveratrol were purchased from Sigma-Aldrich (St Louis, MO, USA). Standards of (–)-epigallocatechin, gallic acid, hydroxytyrosol, malvidin 3-*O*-glucoside chloride, malvidin 3,5-di-*O*-glucoside chloride, procyanidin B2, quercetin 3-*O*-glucuronide, and taxifolin were purchased from Extrasynthese (Geney, France). Standards of caffeic acid, ferulic acid, and vanillic acid were purchased from Fluka (Buchs, Switzerland). Standards of 1-*O*-Galloyl-β-D-glucose and quercetin 3-*O*-glucoside were purchased from PlantMetaChem, Transmit GmbH (Gießen, Germany). Standard of *trans*-piceid was purchased from Selleckchem (Houston, TX, USA).

## Results

### Genetic diversity of polyphenol composition

After elimination of cultivars for which both samples were not available and/or essential data such as berry weight was missing, complete data was obtained for 208 cultivars in 2014, for 161 cultivars in 2015, and for 147 cultivars in both years. The list of samples collected in 2014 and 2015 and their harvest dates is provided in Table S1. Data for all cultivars in both vintages are available in Pinasseau et al. (2017).

Large differences in the phenolic composition were observed between cultivars. Tannins were very abundant in all cultivars with concentrations ranging from 0.4 to 7.5 mg berry^−1^ in 2014, and over 12 mg berry^−1^ in 2015. Anthocyanin contents ranged from less than 1 μg berry^−1^ in white cultivars to 8.5 and 14.7 mg berry^−1^, respectively in 2014 and 2015. Flavonols, and especially quercetin derivatives (quercetin 3-glucoside and quercetin 3-glucuronide), were also abundant, with concentrations ranging from 0.04 to over 6 mg berry^−1^ in 2014 and from 0.06 to over 5 mg berry^−1^ in 2015. Other polyphenol classes were hydroxycinnamic acids (8–2,000 μg berry^−1^) mostly represented by caftaric and coutaric acids, stilbenes (1–745 μg berry^−1^), among which *cis*- and *trans*- piceid and *trans*-resveratrol were the most abundant, dihydroflavonols (trace amounts to 196 μg berry^−1^), and hydroxybenzoic acids (trace amounts to 25 μg berry^−1^). A number of anthocyanin derivatives were also detected. Most of them (i.e., carboxypyranoanthocyanins; e.g., carboxypyranomalvidin 3-glucoside, called vitisin A, caftaric anthocyanin adducts, and series of flavanol-anthocyanin, anthocyanin-flavanol, anthocyanin-ethyl-flavanol, and flavanol-ethyl flavanol adducts), were present in low amounts, except pyranoanthocyanins resulting from reaction of acetaldehyde with anthocyanins, especially pyranomalvidin 3-glucoside (vitisin B), detected at concentrations up to 400 μg berry^−1^.

Table 2 shows the correlation coefficients between irrigated and not irrigated populations, in 2014 and 2015, and between vintages for irrigated and not irrigated samples, for each of the 17 calculated polyphenol composition variables and for berry weight. Berry weight was highly correlated across all four conditions, as expected. The contents (per berry) and concentrations (per g of berry) of all polyphenol families, except flavonols and stilbenes, in irrigated and not-irrigated berries were highly correlated in 2014 but not in 2015. Correlations between years were low under both conditions. In contrast, for all qualitative variables, correlations between irrigated and not irrigated conditions were very high and correlations between years were only slightly lower.

**Table 2:** Stability of polyphenol composition data and berry weight; correlations between irrigated and not irrigated berries in 2014 (2014 I/NI) and 2015 (2015 I/NI), and between 2014 and 2015 berries, under irrigated (I_2014/2015) and not irrigated (NI_2014/2015) conditions.

	**Polyphenol contents per berry**				**Polyphenol concentrations per g berry**			
	**2014 I/NI**	**2015 I/NI**	**I_2014/2015**	**NI_2014/2015**	**2014 I/NI**	**2015 I/NI**	**I_2014/2015**	**NI_2014/2015**
s_acn_n	0.84	0.69	0.58	0.76	0.91	0.68	0.71	0.71
p_acn_acyl	0.82	0.85	0.72	0.76				
p_acn_tri	0.84	0.92	0.86	0.87				
p_acn_met	0.85	0.88	0.83	0.85				
s_acn_n^*^	0.71	0.52	0.32	0.59	0.85	0.52	0.53	0.56
p_acn_acyl^*^	0.92	0.94	0.88	0.91				
p_acn_tri^*^	0.95	0.96	0.95	0.94				
p_acn_met^*^	0.94	0.91	0.94	0.91				
s_tann	0.81	0.29	0.30	0.22	0.76	0.43	0.44	0.48
dp_tann	0.89	0.96	0.69	0.72				
p_tann_gall	0.78	0.92	0.52	0.63				
p_tann_tri	0.78	0.93	0.65	0.72				
s_flavo	0.47	0.59	0.25	0.20	0.47	0.50	0.20	0.03
p_flavo_mono	0.82	0.80	0.68	0.62				
p_flavo_di	0.75	0.71	0.50	0.47				
p_flavo_tri	0.90	0.90	0.88	0.83				
p_flavo_met	0.89	0.83	0.84	0.81				
p_flavo_glucur	0.63	0.72	0.48	0.37				
s_ahyb	0.78	0.33	0.52	0.41	0.80	0.26	0.49	0.43
s_ahyc	0.82	0.58	0.64	0.55	0.76	0.52	0.62	0.58
s_stil	0.50	0.30	0.32	0.39	0.56	0.20	0.32	0.34
berry weight	0.91	0.88	0.87	0.84				

* *Calculated with colored (black, red, and pink) cultivars only*.

**Figure 1:**
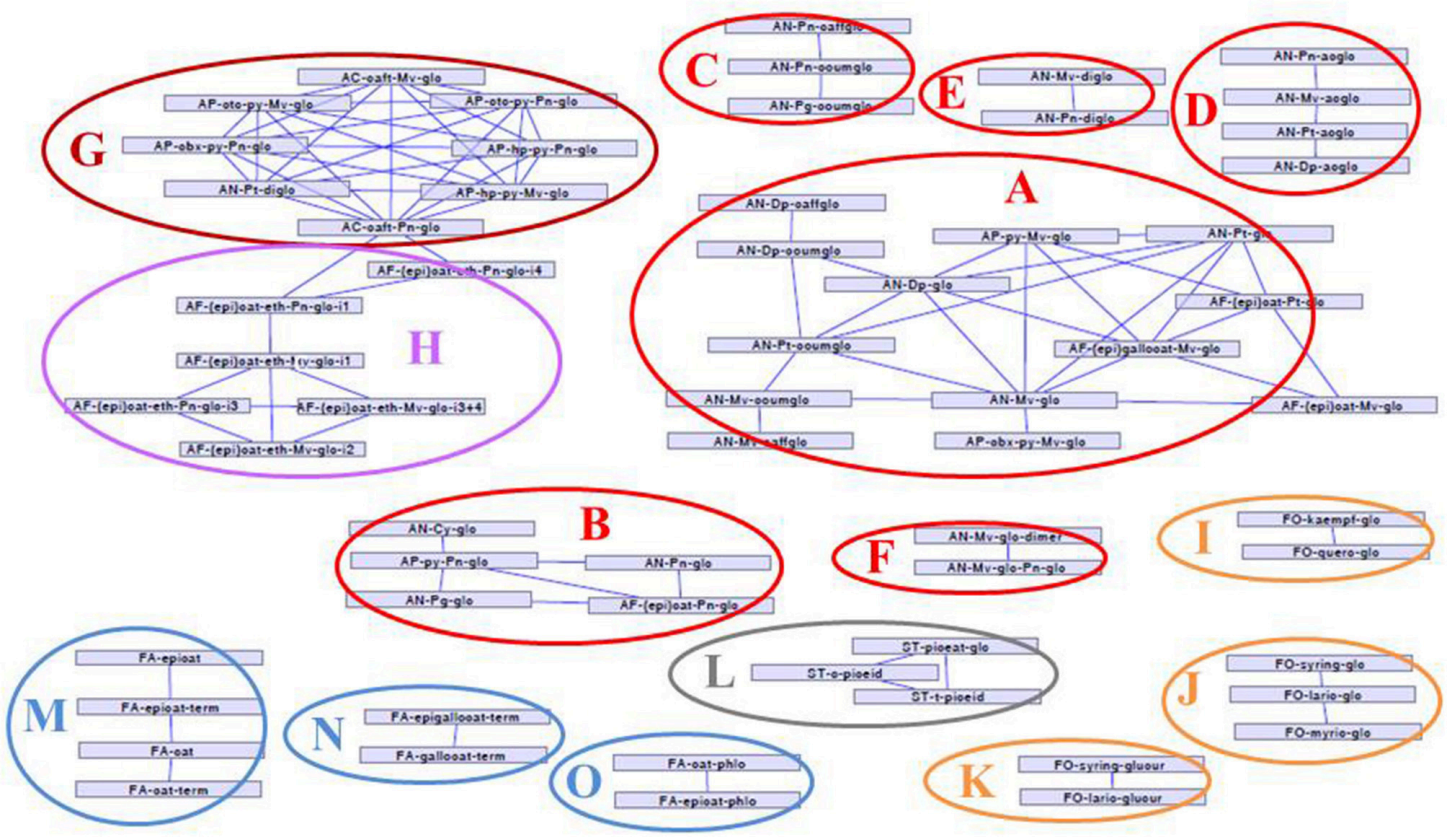
Correlation network (correlation values >0.8) established from the 105 MRM polyphenol composition variables (in mg berry^−1^, coded as in Table 1) on the whole data set (2014 and 2015). Clusters of the different polyphenol groups are colored differently: anthocyanins (red), anthocyanin derived pigments (dark red and purple), flavonols (yellow), flavan-3-ols (blue), stilbenes (gray).

Correlation networks established from the phenolic composition data showed several clusters. Correlations >0.8 are presented in Figure 1. The content of malvidin 3-glucoside was correlated on one hand with those of delphinidin 3-glucoside, petunidin-3-glucoside, and of their coumaroyl and caffeoyl derivatives and, on the other hand, with those of some anthocyanin derivatives [pyranomalvidin 3-glucoside, carboxypyranomalvidin 3-glucoside, (epi)gallocatechin-malvidin 3-glucoside, (epi)catechin-malvidin 3-glucoside, and (epi)catechin-petunidin 3-glucoside] (Figure 1, **A**). Pelargonidin, cyaniding, and peonidin 3-glucosides were correlated together and with peonidin derivatives, namely pyranopeonidin 3-glucoside and (epi)catechin–peonidin 3-glucoside (**B**) while their caffeoyl and p-coumaroyl esters formed another group (**C**). All acetylated anthocyanins were correlated together in a separate cluster (**D**). Other types of anthocyanin pigments, namely anthocyanin 3,5-di-*O*-glucosides (**E**), anthocyanin dimers (**F**), phenylpyranoanthocyanins correlated between them and with caftaric-anthocyanin adducts (**G**) and the different isomers of (epi)catechin-ethyl-peonidin−3-glucoside and (epi)catechin-ethyl-malvidin 3-glucoside (**H**) formed additional groups. Flavonols clustered in three different groups consisting of kaempferol and quercetin 3-glucosides (**I**), myricetin, laricitrin, and syringetin 3-glucosides (**J**), and laricitrin and syringetin 3-glucuronides (**K**), respectively. Stilbene glucosides (*cis*- and *trans*- piceids and piceatannol glucoside) formed another correlation network (**L**). Flavan-3-ol variables formed three clusters: (epi)catechin monomers and terminal units (**M**), (epi)gallocatechin terminal units (**N**), and (epi)catechin phloroglucinol derivatives (extension and upper units in the tannin structures) (**O**).

### Vine water status in 2014 and 2015

**Figure 2:**
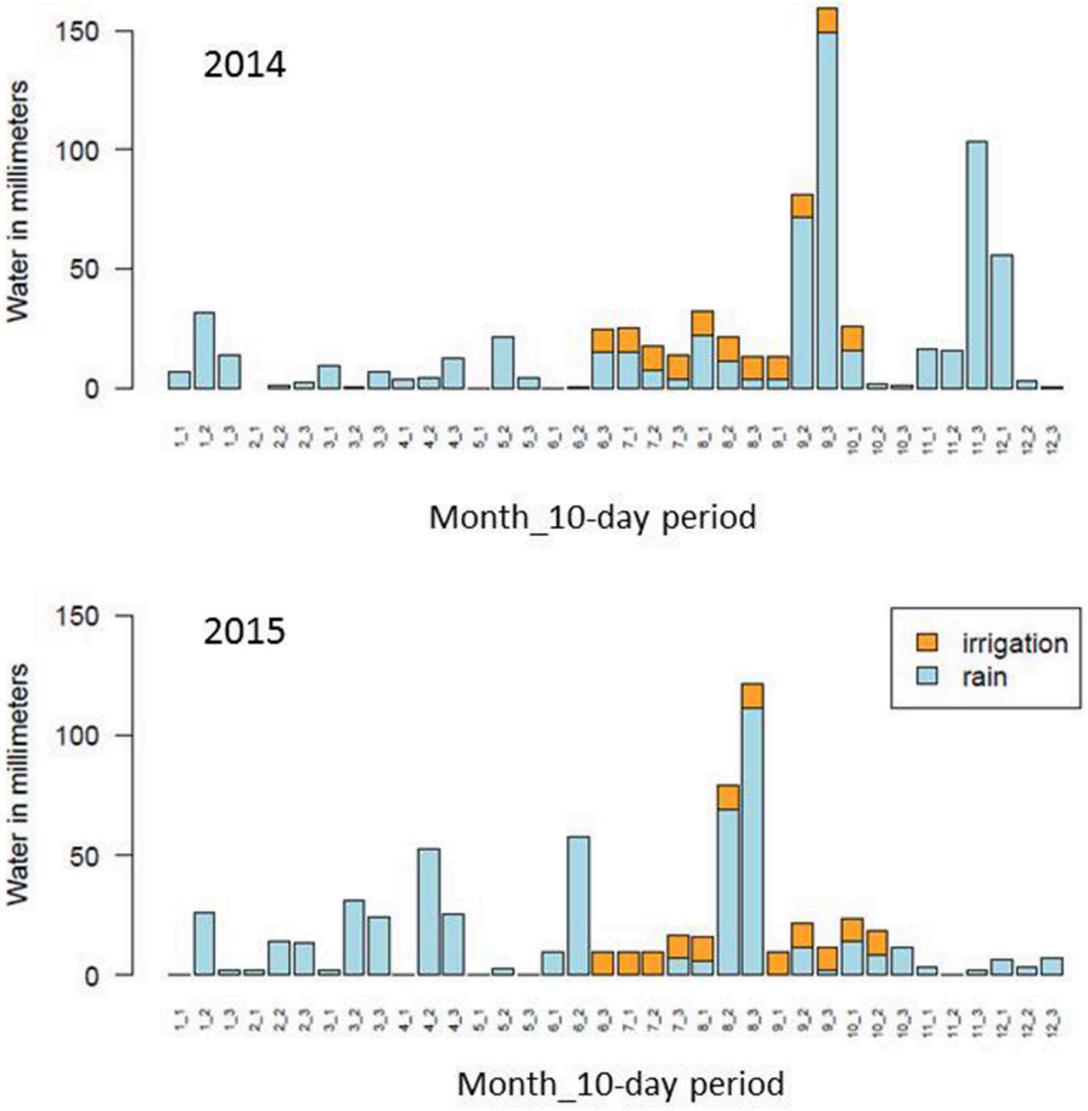
Bar plots showing water quantities supplied by rainfall and irrigation.

Information from the rain and irrigation data and from the measures of δ^13^C and berry weight was combined to characterize the vine water status during the vegetative seasons 2014 and 2015. Bar plots showing water quantities supplied by rainfall and irrigation are provided in Figure 2, showing that the total quantity of rainfall received within the plot trial the preceding winter and spring was very different After including data from 2013 (data not shown), the total rainfall received from November to the second third of June (before irrigation started) was 187.5 and 460.5 mm for 2014 and 2015, respectively. Another notable difference between the two vegetative seasons was the earlier occurrence of summer rainfall in 2015 as compared to 2014 (Figure 2).

### Cultivar response to water stress in vintages 2014 and 2015

**Figure 3:**
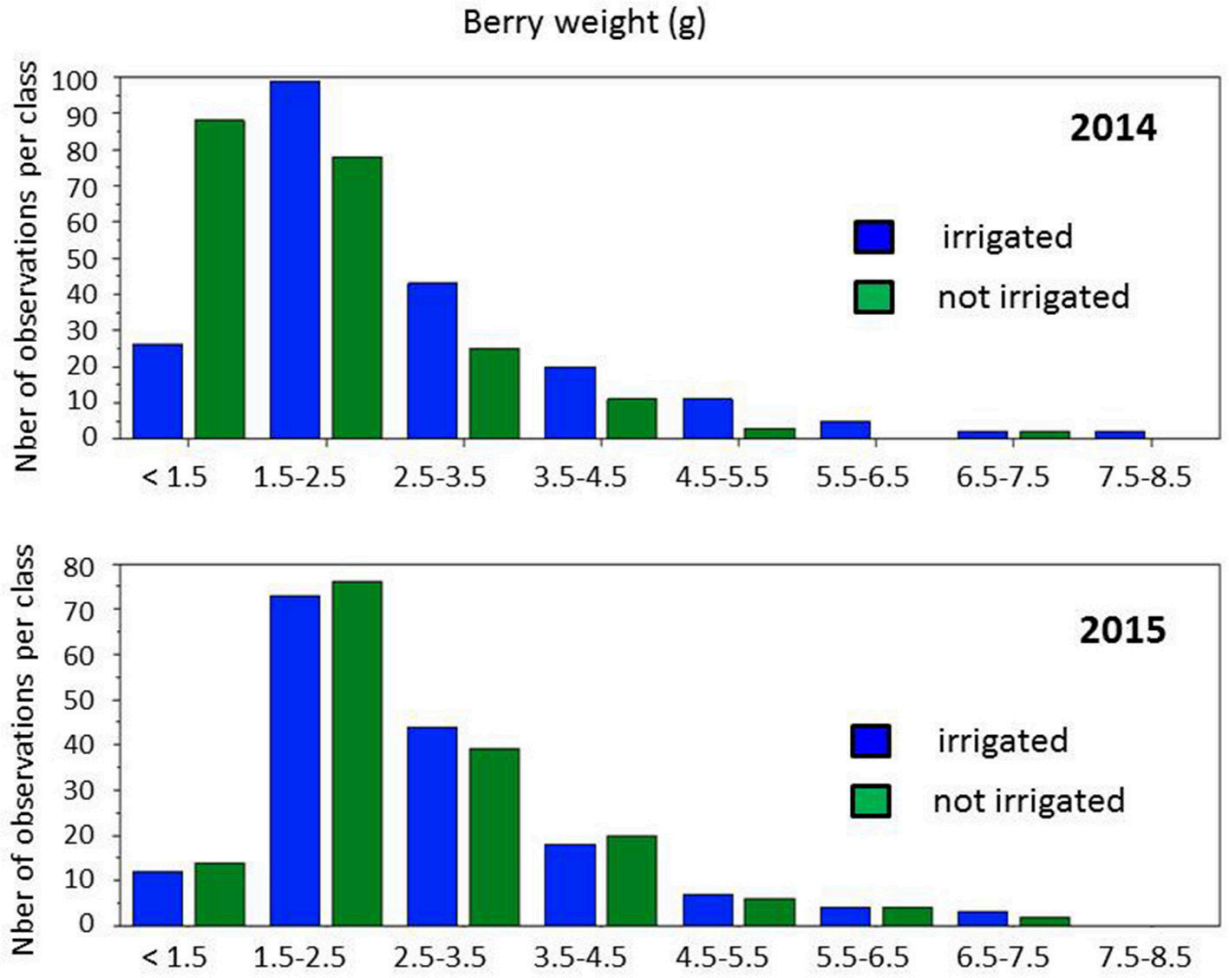
Distribution of berry weights in the population grown with and without irrigation, in 2014 and 2015.

**Figure 4:**
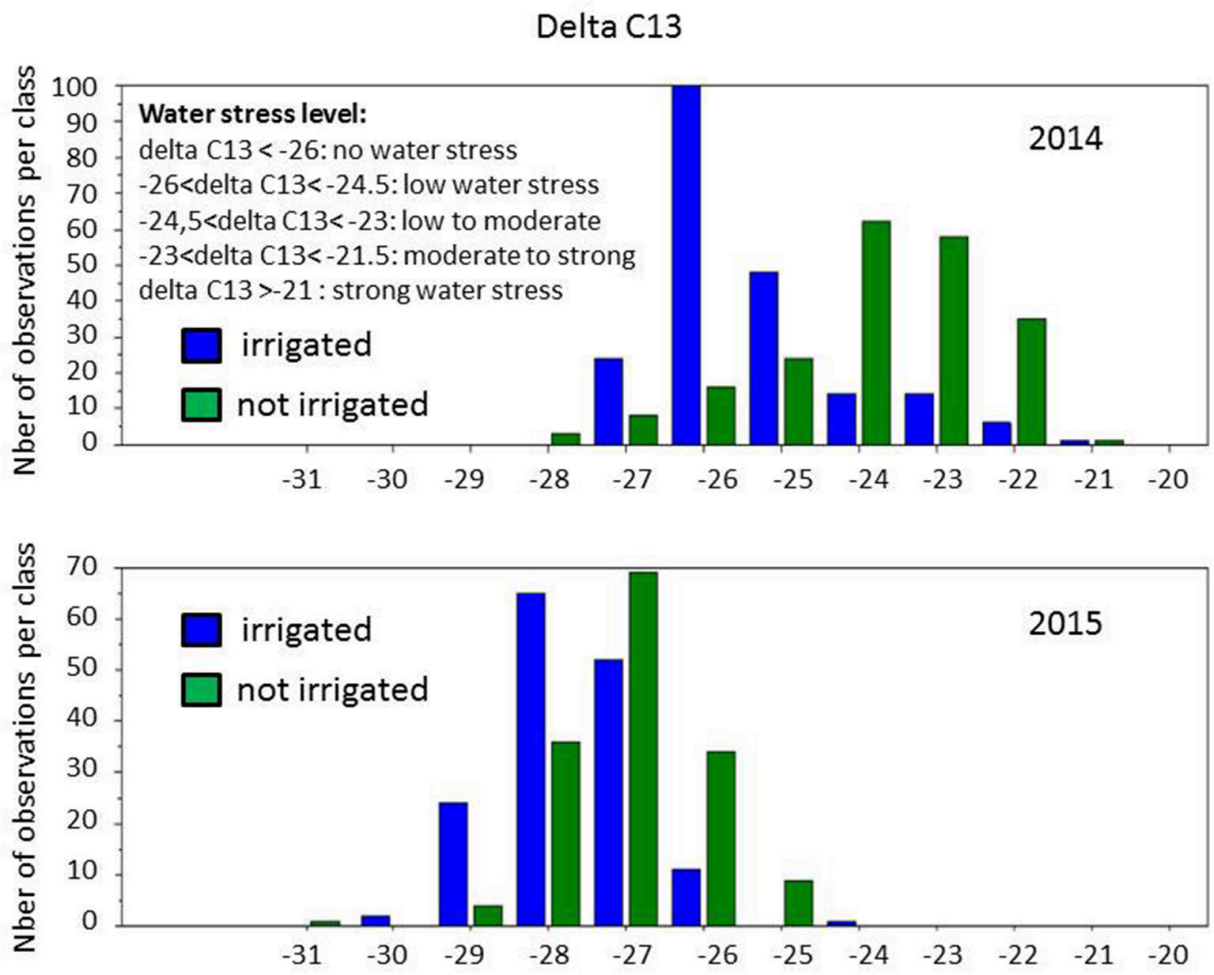
Distribution of δ^13^C values in the population grown with and without irrigation, in 2014 and 2015.

A first round of statistical analysis was performed with one-way ANOVA analysis on the four data sets (irrigated and not-irrigated, 2014 and 2015) available for 147 cultivars (Table S2). The absence of significant differences (at *p* = 0.05) in refractive index values between conditions in both years confirmed that berries were actually collected at the same developmental stages, while differences between years indicated a slight vintage effect. However, large phenotypic diversity was observed on berry weight (Figure 3). Water deficit induced a slight shift toward smaller berry size in 2014, with the major class below 1.5 g and between 1.5 and 2.5 g per berry, respectively, in non-irrigated and irrigated berries. Distribution of berry sizes was not impacted by irrigation in 2015. Large variations were also observed for δ^13^C values within the collection (Figure 4). Irrigation induced larger shifts in 2014 than in 2015 and the whole population showed much lower values in 2015 than in 2014, regardless of the irrigation regime. Berry weight was significantly lower in not-irrigated berries in 2014 but not in 2015. Irrigation induced significant differences on the δ^13^C values in both vintages, but water stress was much lower in 2015with δ^13^C values significantly higher than in 2014. Taken together, these data indicate that irrigation induced a marked contrast in 2014 but a very limited one in 2015.

ANOVA analysis performed on the 105 polyphenol variables expressed in mg per g of berry (Table S2) showed that most tannins and flavonols and of their total concentrations were significantly reduced by irrigation in 2014 but not in 2015. In 2015, the concentrations of *cis*-resveratrol and piceatannol were significantly increased by irrigation and that of glucogallin was significantly reduced. Significant vintage effect was also found on over 50 compounds, with significantly higher levels in 2015 for the majority of them, except gallocatechin and epigallocatechin which were more abundant in 2014.

When the analysis was performed on the data expressed per berry (Table S2), no significant difference was found in the levels of phenolic compounds between irrigated and not irrigated conditions in 2015 whereas seven compounds from the flavan-3-ol family and oxidized glutathione were significantly increased by irrigation in 2014. Numerous compounds, distributed within all polyphenol families, were significantly higher in 2015 than in 2014, as well as total flavan-3-ols, flavonols, hydroxybenzoic acids, and hydroxycinnamic acids.

One way ANOVA was also performed separately on the complete 2014 and 2015 data sets (Table 3). There was no statistically significant difference between irrigated and not-irrigated conditions (at *p* = 0.05) in 2015 on polyphenol composition. In contrast, in 2014, irrigation induced significant changes in the content (per berry) of 16 polyphenols and in the concentration (per g of berry) of 47 compounds.

**Table 3:** Results of the ANOVA performed on the data of irrigated (I) and non-irrigated (NI) vines separately on 2014 and 2015; polyphenol composition data in and microgram per berry and microgram per g of berry; variable codes are provided in Table 1.

**Variable Code^a^**	**Content (μg/berry)**						**Concentration (μg/g berry)**					
	**2014 (208 cultivars)**			**2014 (208 cultivars)**			**2015 (161 cultivars)**					
	**NI**	**IR**	**SNK**	**NI**	**IR**	**SNK**	**NI**	**IR**	**SNK**	**NI**	**IR**	**SNK**
**POLYPHENOLIC COMPOSITION**												
AN-Pg-glc	0.67	0.59		0.87	0.96		0.40	0.26	^*^	0.25	0.41	
AN-Cy-glc	56.79	53.36		71.77	81.11		32.05	22.03		28.83	36.27	
AN-Dp-glc	25.78	22.20		30.25	30.82		18.04	11.66	^*^	14.66	15.02	
AN-Pt-glc	27.33	24.60		34.26	32.67		19.15	12.74	^*^	16.26	15.78	
AN-Pn-glc	58.77	57.99		63.17	70.35		36.12	25.68		28.21	32.25	
AN-Mv-glc	194.41	179.54		219.41	195.34		135.03	89.40	^*^	101.48	90.94	
AN-Cy-diglc	0.24	0.23		0.34	0.40		0.14	0.09	^*^	0.13	0.17	
AN-Dp-diglc	0.28	0.22		0.26	0.26		0.18	0.11		0.12	0.12	
AN-Pt-diglc	0.03	0.04	^*^	0.14	0.15		0.02	0.01	^*^	0.058	0.061	
AN-Pn-diglc	0.06	0.06		0.23	0.19		0.03	0.03		0.088	0.074	
AN-Mv-diglc	0.11	0.10		1.12	0.61		0.07	0.05		0.37	0.20	
AN-Pg-acglc	0.06	0.06		0.15	0.16		0.04	0.03		0.064	0.068	
AN-Cy-acglc	2.75	3.00		2.79	3.43		1.95	1.62		1.38	1.79	
AN-Dp-acglc	7.69	7.84		7.68	8.73		6.01	4.64		4.25	4.66	
AN-Pt-acglc	7.85	8.23		8.33	8.83		6.12	4.71		4.46	4.78	
AN-Pn-acglc	10.33	10.34		11.65	12.37		7.21	5.42		5.87	6.32	
AN-Mv-acglc	63.98	69.97		74.50	67.48		47.03	37.54		36.94	35.11	
AN-Pg-coumglc	0.27	0.27		0.39	0.37		0.18	0.13		0.16	0.16	
AN-Cy-coumglc	11.31	11.77		17.45	17.38		6.63	5.26		7.26	7.79	
AN-Dp-coumglc	31.29	29.68		50.78	44.75		22.15	15.47		23.21	21.39	
AN-Pt-coumglc	26.99	26.40		42.65	34.96		19.08	13.37		19.14	16.44	
AN-Pn-coumglc	35.80	36.63		47.21	48.91		21.84	16.52		20.72	22.00	
AN-Mv-coumglc	185.94	191.62		306.18	230.11		128.94	91.66		132.17	102.46	
AN-Cy-caffglc	0.09	0.11		0.19	0.22		0.054	0.04		0.08	0.09	
AN-Dp-caffglc	0.07	0.09		0.20	0.21		0.05	0.05		0.09	0.092	
AN-Pt-caffglc	0.11	0.12		0.21	0.20		0.07	0.05		0.09	0.09	
AN-Pn-caffglc	0.39	0.42		0.55	0.65		0.26	0.20		0.24	0.29	
AN-Mv-caffglc	0.99	1.18		1.89	1.67		0.69	0.57		0.83	0.76	
AN-Mv-glc-Pn-glc	0.08	0.09		0.15	0.16		0.05	0.04		0.063	0.068	
AN-Mv-glc-dimer	0.08	0.08		0.15	0.16		0.05	0.04		0.062	0.068	
AP-py-Pn-glc	3.05	2.30		3.89	4.44		2.01	1.08	^*^	1.65	2.11	
AP-py-Mv-glc	13.05	9.00		19.86	17.57		9.42	5.30	^*^	9.18	8.46	
AP-hp-py-Pn-glc	0.03	0.04	^*^	0.14	0.15		0.02	0.02	^*^	0.06	0.06	
AP-hp-py-Mv-glc	0.04	0.044	^*^	0.14	0.15		0.02	0.02	^*^	0.06	0.06	
AP-ctc-py-Pn-glc	0.04	0.04	^*^	0.11	0.11		0.02	0.02	^*^	0.04	0.05	
AP-ctc-py-Mv-glc	0.04	0.04	^*^	0.11	0.11		0.02	0.02	^*^	0.04	0.05	
AP-cbx-py-Pn-glc	0.04	0.04		0.15	0.15		0.02	0.02	^*^	0.06	0.06	
AP-cbx-py-Mv-glc	0.32	0.34		0.45	0.41		0.22	0.15		0.19	0.17	
AT-Pn-glc-epi-gallocat	0.07	0.08		0.14	0.16		0.05	0.04		0.06	0.07	
AT-Pn-glc-epi-cat	0.16	0.17		0.19	0.20		0.11	0.08		0.08	0.09	
AT-Mv-glc-epi-gallocat	0.08	0.08		0.14	0.17		0.051	0.04		0.06	0.067	
AT-Mv-glc-epi-cat	0.087	0.096		0.24	0.19		0.05	0.03		0.07	0.07	
AT-epi-gallocat-Pn-glc	0.15	0.13		0.55	0.52		0.1	0.06	^*^	0.24	0.23	
AT-epi-gallocat-Mv-glc	0.27	0.24		0.54	0.45		0.18	0.12	^*^	0.24	0.20	
AT-epi-cat-Pn-glc	0.11	0.11		0.31	0.33		0.06	0.04		0.13	0.14	
AT-epi-cat-Mv-glc	0.19	0.16		0.68	0.59		0.12	0.07	^*^	0.30	0.27	
AT-epi-cat-eth-Pn-glc-i1	0.039	0.045		0.14	0.15		0.02	0.02		0.06	0.06	
AT-epi-cat-eth-Pn-glc-i2	0.052	0.086		0.14	0.17		0.03	0.04		0.06	0.08	
AT-epi-cat-eth-Pn-glc-i3	0.077	0.25		0.18	0.23		0.04	0.1		0.08	0.09	
AT-epi-cat-eth-Pn-glc-i4	0.048	0.070		0.14	0.15		0.03	0.03		0.06	0.06	
AT-epi-cat-eth-Mv-glc-i1	0.054	0.081		0.19	0.18		0.03	0.04		0.08	0.07	
AT-epi-cat-eth-Mv-glc-i2	0.07	0.13		0.30	0.33		0.04	0.06		0.11	0.13	
AT-epi-cat-eth-Mv-glc-i3+4	0.18	0.49	^*^	0.76	0.48		0.12	0.24		0.26	0.18	
AC-caft-Pn-glc	0.035	0.041	^*^	0.16	0.15		0.02	0.02	^*^	0.06	0.06	
AC-caft-Mv-glc	0.037	0.043	^*^	0.14	0.15		0.02	0.02	^*^	0.06	0.06	
HF-taxif	0.68	0.82		1.83	1.80		0.38	0.33		0.76	0.76	
HF-astilb	2.55	3.72		5.02	4.74		1.47	1.62		1.86	1.70	
FO-syring-glucur	0.040	0.043		0.09	0.09		0.03	0.02	^*^	0.036	0.037	
FO-syring-glc	8.97	9.68		11.47	7.70		6.02	4.66		4.98	3.29	
FO-querc-glucur	79.49	80.57		97.92	100.50		43.67	31.32	^*^	39.74	42.01	
FO-querc-glc	245.77	242.26		344.07	327.16		126.35	90.73	^*^	126.60	129.51	
FO-myric-glucur	0.33	0.28		0.34	0.27		0.20	0.12	^*^	0.14	0.12	
FO-myric-glc	13.72	12.96		21.38	17.48		8.85	6.22	^*^	9.14	7.56	
FO-laric-glucur	0.061	0.056		0.10	0.10		0.04	0.03	^*^	0.04	0.04	
FO-laric-glc	9.73	10.15		11.45	8.28		6.42	4.92		4.93	3.55	
FO-kaempf-glucur	0.18	0.17		0.13	0.13		0.09	0.06	^*^	0.05	0.05	
FO-kaempf-glc	182.92	197.16		230.08	211.33		93.72	71.85	^*^	80.63	79.71	
FO-isorham-glucur	0.12	0.11		0.13	0.12		0.07	0.05	^*^	0.06	0.05	
FO-isorham-glc	21.24	21.14		18.78	16.15		12.05	8.91	^*^	7.59	6.48	
ST-c-resver	0.20	0.21		0.22	0.25		0.12	0.08	^*^	0.091	0.11	
ST-t-resver	5.69	6.13		8.60	9.73		3.57	2.54		3.36	4.02	
ST-c-piceid	24.63	24.56		28.37	27.75		15.54	9.78	^*^	12.19	11.98	
ST-t-piceid	6.26	7.02		8.98	8.95		3.87	2.90		3.66	3.59	
ST-piceat-glc	0.70	0.91		1.19	1.26		0.45	0.35		0.51	0.53	
ST-piceat	0.25	0.33	^*^	0.53	0.58		0.16	0.13		0.22	0.27	
ST-resver-dimer	0.99	1.18		1.50	1.43		0.63	0.50		0.68	0.65	
FA-gallocat	3.13	3.80		1.07	1.01		1.82	1.61		0.44	0.44	
FA-epigallocat	1.01	1.22		0.84	0.88		0.63	0.57		0.37	0.43	
FA-epicat	2.99	4.52	^*^	3.96	4.50		1.62	1.76		1.62	1.99	
FA-cat	11.57	16.72	^*^	22.74	25.36		6.00	6.37		8.71	10.68	
FA-epicat-eth-epicat-i1	0.041	0.049		0.14	0.15		0.025	0.022		0.06	0.06	
FA-epicat-eth-epicat-i2+3	0.14	0.16		0.30	0.24		0.098	0.080		0.13	0.11	
FA-gallocat-term	28.23	30.43		30.11	26.26		15.59	12.38	^*^	11.94	11.21	
FA-epigallocat-term	5.26	5.40		4.93	4.47		3.05	2.30	^*^	2.05	1.98	
FA-epicat-term	8.35	10.48	^*^	12.93	13.69		4.59	4.17		5.29	5.92	
FA-epicat-gall-term	3.75	3.87		5.64	5.47		2.08	1.56	^*^	2.27	2.28	
FA-cat-term	87.11	116.11	^*^	183.85	191.79		46.53	45.39		72.18	79.60	
FA-epigallo-gallocat-phlo	874.66	894.32		1109.09	979.30		513.98	382.53	^*^	469.47	438.24	
FA-epicat-phlo	1604.34	1897.39	^*^	2368.61	2399.51		899.24	771.81	^*^	992.33	1048.95	
FA-epicat-gall-phlo	96.59	105.63		108.94	109.48		55.18	43.82	^*^	45.60	48.33	
FA-cat-phlo	7.84	9.23	^*^	16.04	16.08		4.40	3.74	^*^	6.48	6.92	
HB-glucogall	0.68	0.68		1.84	1.52		0.38	0.27	^*^	0.73	0.61	
HB-vanill-ac	0.10	0.11		0.24	0.28		0.064	0.046		0.10	0.12	
HB-syring-ac	0.33	0.32		0.87	0.78		0.23	0.15	^*^	0.40	0.34	
HB-protocat-ac	0.15	0.15		0.44	0.50		0.088	0.061	^*^	0.18	0.21	
HB-gall-ac	0.27	0.26		0.74	0.73		0.17	0.11	^*^	0.31	0.31	
HC-ct-coutar-ac	80.54	90.26		113.29	113.21		44.04	36.32	^*^	46.81	48.60	
HC-ct-caftar-ac	122.00	140.83		229.55	228.92		69.21	57.68	^*^	94.99	97.34	
HC-t-fertar-ac	4.56	4.38		10.91	10.27		2.49	1.72	^*^	4.33	4.19	
HC-t-caffeic-ac	0.04	0.05	^*^	0.15	0.15		0.022	0.021		0.06	0.06	
HC-t-coumar-ac	0.066	0.067		0.19	0.20		0.039	0.029	^*^	0.08	0.08	
HC-t-ferul-ac	0.040	0.044		0.16	0.17		0.023	0.019	^*^	0.07	0.07	
OT-OH-tyrosol	0.071	0.074		0.15	0.16		0.040	0.031	^*^	0.06	0.07	
OT-GSSG	1.74	2.13		3.10	3.21		0.95	0.90	^*^	1.32	1.36	
OT-GSH	4.82	5.24		9.30	10.40		2.74	2.35		3.92	4.70	
**POLYPHENOLIC INDICES CALCULATED FROM THE PREVIOUS RESULTS**												
s_AN_n	750.39	736.69		994.54	893.37		509.54	359.35	^*^	447.55	415.62	
s_FO	562.55	574.57		735.92	689.30		297.51	218.89	^*^	273.95	272.40	
s_FA	2716.13	3072.86	^*^	3840.13	3746.05		1544.63	1267.71	^*^	1607.61	1643.40	
s_HB	1.53	1.52		4.13	3.81		0.93	0.64	^*^	1.72	1.60	
s_HC	207.25	235.63		354.26	352.92		115.83	95.79	^*^	146.35	150.35	
s_ST	38.73	40.34		49.38	49.94		24.34	16.29	^*^	20.70	21.15	
p_AN_acyl (%)	38.88	41.91		47.70	46.84							
p_AN_tri (%)	56.69	56.32		57.02	55.25							
p_AN_met (%)	56.39	56.83		58.14	57.51							
p_FO_mono (%)	29.03	27.84		26.98	25.70							
p_FO_di (%)	62.84	63.77		65.81	67.29							
p_FO_tri (%)	8.13	8.39		7.21	7.01							
p_FO_met (%)	8.55	8.40		6.04	5.57							
p_FO_glucur (%)	16.97	18.00		18.43	19.73							
p_FA_tri (%)	34.17	30.71	^*^	28.52	25.54	^*^						
p_FA_gall (%)	3.63	3.51		3.14	3.25							
dp_FA	23.10	21.04	^*^	19.40	18.73							
**OTHER PARAMETERS**												
deltaC13^b^	−23.812	−25.392	^*^	−26.95	−27.689	^*^						
brix^b^	20.01	19.70		19.34	19.33							
berry weight (g)	1.93	2.67	^*^	2.67	2.73							

a *Variable codes as in Table 1*.

* *SNK: results of the Student-Newman-Keuls grouping (p < 0.05)*.

b *A few missing values have been removed from the calculation*.

Taken together, these results indicate that berries were probably not exposed to any sufficient water stress regime in 2015 to induce changes in their phenolic composition. Consequently, data from 2015 were not further explored in this study.

### Impact of water stress on polyphenol composition

**Figure 5:**
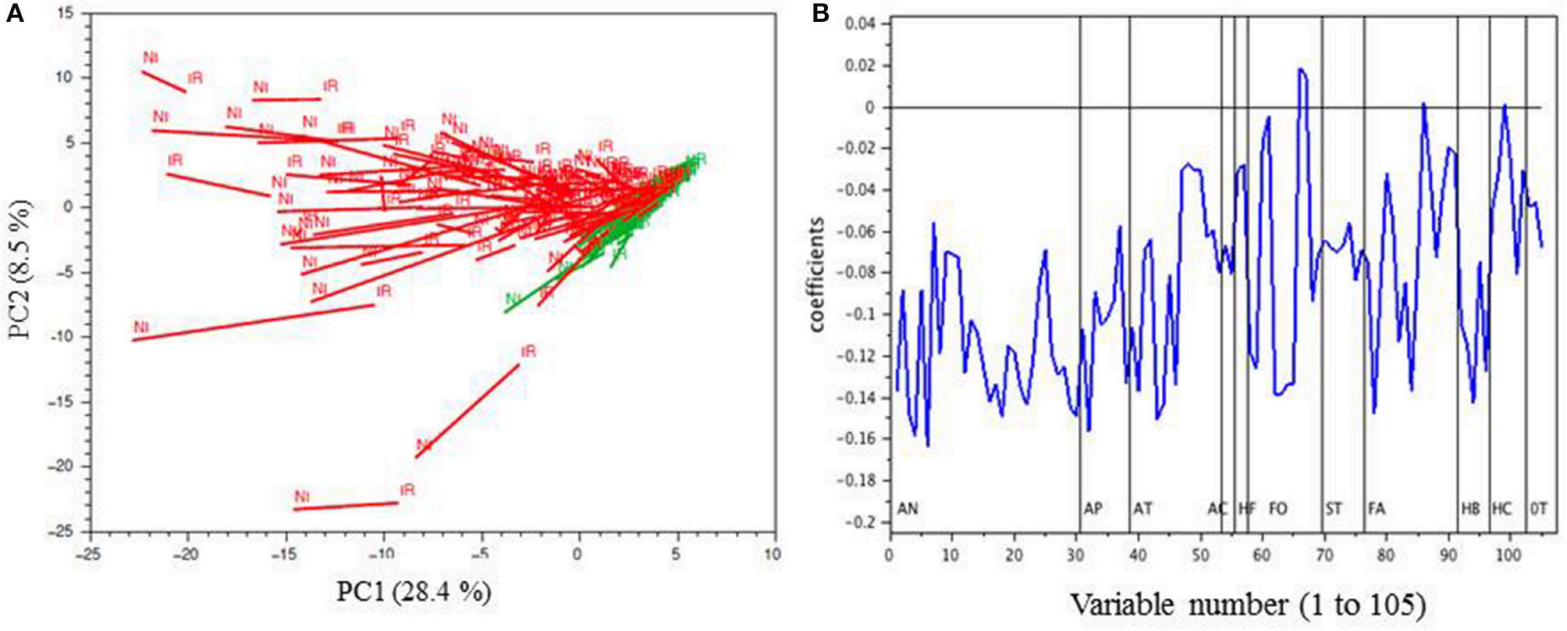
PCA of the MRM phenolic composition data of berry skin samples collected in 2014 (mg g^−1^); **(A)**, projection of the samples on PC1 and PC2; red and white cultivars are represented in red and in green, respectively; IR, irrigated, NI, not-irrigated. **(B)**, loadings of the variables (coded as in Table 1) on PC1. AN, native anthocyanins+dimers; AP, pyrano anthocyanins; AF, anthocyanin-flavanol adducts; AC, caftaric-anthocyanin adducts; HF, dihydroflavonols; FO, flavonols; ST, stilbenes; FA, flavanols (tannins); HB, hydroxybenzoic acids; HC, hydroxycinnamic acids; OT, others.

Principal component analysis (PCA) was performed on the phenolic composition data of all berry skin samples collected in 2014, expressed in mg per g of fresh berry. Projection of the samples on the first two principal components, accounting together for 37% of the variance, showed large cultivar differences, as well as a strong impact of irrigation (Figure 5A). White and red cultivars were separated along the first axis which was negatively correlated with the concentrations of most phenolic compounds, including anthocyanins, especially delphinidin, petunidin, and malvidin 3-glucosides, myricetin, laricitrin, and syringetin glycosides, hydroxybenzoic acids, especially gallic and syringic acids, and epigallocatechin, both in the free form and as terminal units of proanthocyanidins (Figure 5B). Non-irrigated samples generally appeared shifted negatively along the first axis, indicating that they contained higher levels of these molecules.

ANOVA analysis of variance performed on the polyphenol composition data set expressed per g berry (Table 3) indicated that berries from irrigated vines contained significantly lower concentrations of the *cis* isomers of resveratrol and piceid, of all tannin units determined after phloroglucinolysis, and of most benzoic acids, hydroxycinnamic acids, and flavonols. The concentrations of some anthocyanins, namely 3-glucosides of pelargonidin, delphinidin, petunidin and malvidin, cyanidin 3,5-diglucoside and petunidin 3,5-diglucoside were also significantly decreased, as well as those of some anthocyanin derivatives, namely pyranoanthocyanins, tannin-anthocyanin adducts, and caftaric anthocyanin adducts. Other variables such as the concentrations of flavan-3-ol monomers were not significantly modified.

**Figure 6:**
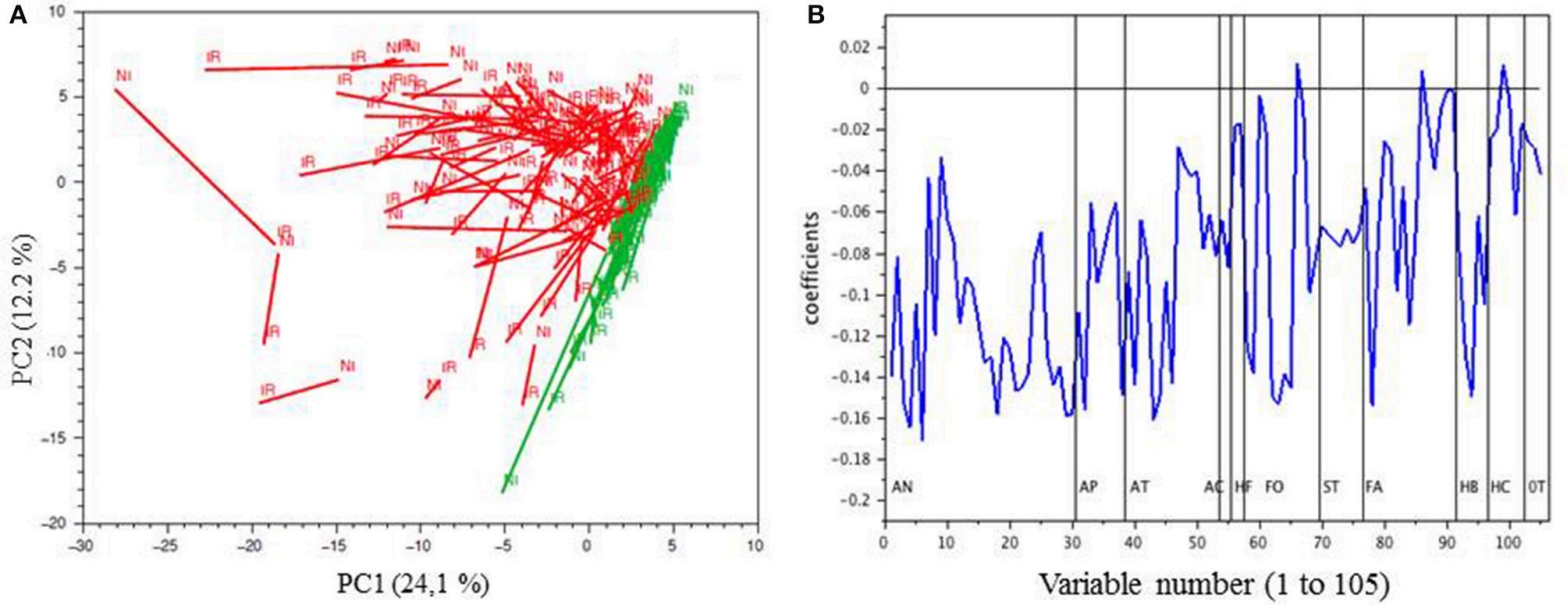
PCA of the MRM phenolic composition data of berry skin samples collected in 2014 (mg berry^−1^); **(A)**, projection of the samples on PC1 and PC2; red and white cultivars are represented in red and in green, respectively; IR, irrigated; NI, not-irrigated. **(B)**, loadings of the variables (coded as in Table 1) on PC1. AN, native anthocyanins+dimers; AP, pyrano anthocyanins; AF, anthocyanin-flavanol adducts; AC, caftaric-anthocyanin adducts; HF, dihydroflavonols; FO, flavonols; ST, stilbenes; FA, flavanols (tannins); HB, hydroxybenzoic acids; HC, hydroxycinnamic acids; OT, others.

When PCA was performed on the phenolic composition data expressed per berry (Figure 6A), most samples appeared shifted along the first and/or second axis, but in different directions. Again, white cultivars were separated from red cultivars along the first axis, which was negatively associated with the same phenolic compounds as in the previous PCA (Figure 6B).

When the data was expressed per berry, 16 compounds were significantly increased in berries from irrigated samples (Table 3). Thus, water stress induced a significant decrease of the biosynthesis of catechin and epicatechin, both as flavan-3-ol monomers and as constitutive units of proanthocyanidins, total flavan-3-ols, phenyl- and catechyl-pyranoanthocyanins, caftaric-anthocyanin adducts, (epi)catechin-ethyl-malvidin-3- glucoside, caffeic acid, and piceatannol. Moreover, some qualitative flavan-3-ol variables, namely tannin mDP, and % trihydroxylated tannin units were significantly reduced by irrigation.

**Figure 7:**
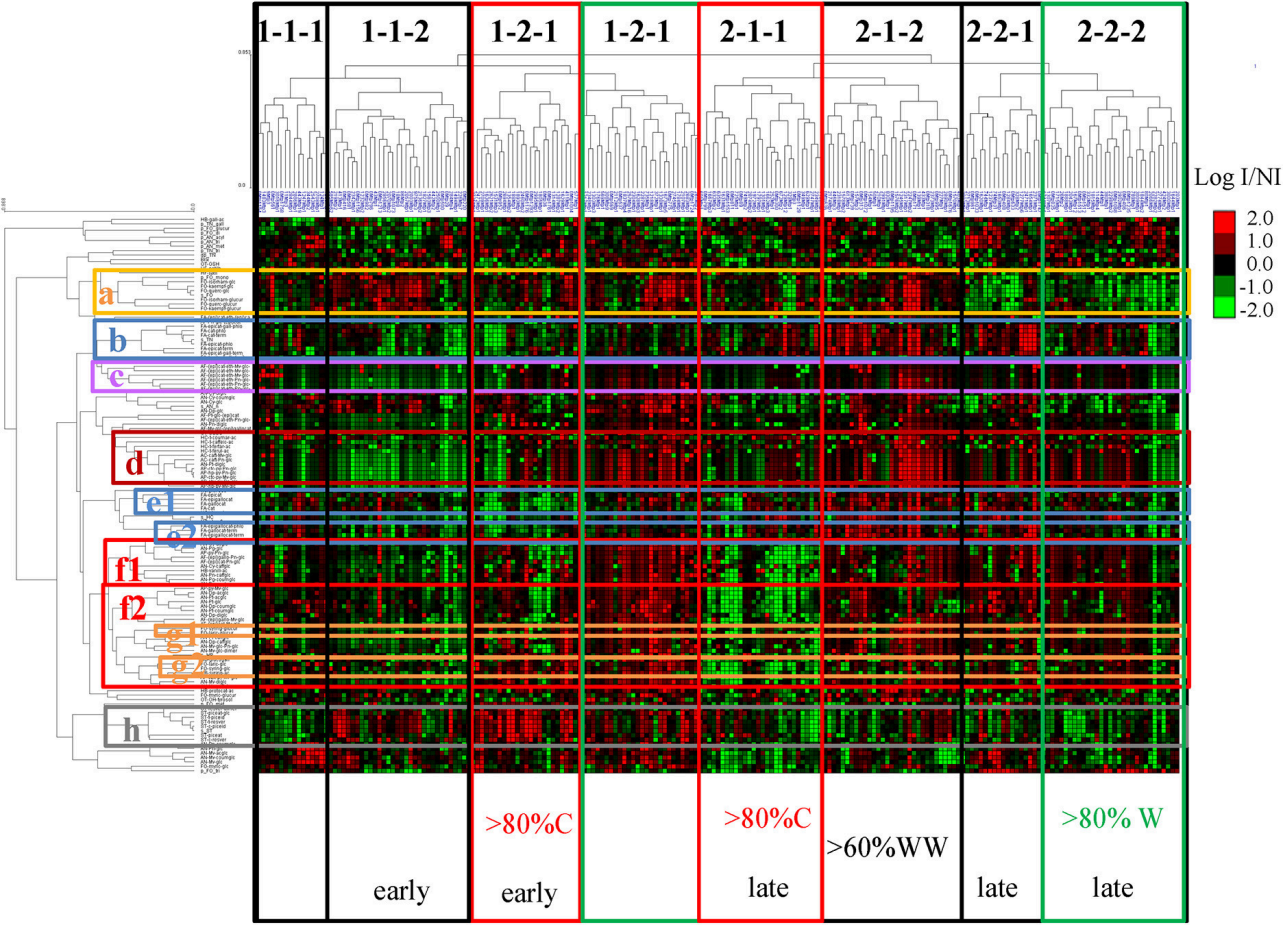
Unsupervised hierarchical clustering of metabolites and cultivars affected by drought; normalized lines (centered and reduced) of Log (content I/content NI), calculated for all variables, with polyphenol concentrations expressed in mg berry^−1^, on the 2014 data set. Codes for variables and cultivars are provided in Table 1 and Table S2, respectively. Clusters of the different polyphenol groups are colored differently: anthocyanin (red; **f1:** mono hydroxylated and **f2:** di-hydroxylated), anthocyanin derived pigments (purple; **c**), hydroxycinnamic acids and their anthocyanin derivatives(dark red; **d**).flavonols and dihydroflavonols (yellow; **a:** mono- and di-hydroxylated; **g1** and **g2:** trihydroxylated), flavan-3-ols (blue; **b:** tannin subunits and sum of flavan-3-ols; **e1** and **e2:** flavan-3-ol monomers and terminal units), stilbenes (gray; **h**). Subgroups of cultivars (1-1-1, 1-1-2, …) and significantly different distribution of colors (mostly colored: C, in red; mostly White: W, in green), genetic origin (WW) and precocity (early, late) in individual subgroups compared to the entire population (see Figures 9, 10, and Table S3) are also illustrated.

Unsupervised hierarchical clustering of metabolites and cultivars affected by drought was performed on the response of polyphenol composition to water status, with data expressed as log (irrigated/non-irrigated), with polyphenol contents expressed per berry. The resulting plot (Figure 7) shows different response patterns for different cultivars and for the different groups of analytical variables. Groups of compounds whose content varies in the same direction in response to irrigation can be distinguished. Cluster **a** contained mainly mono- and di-hydroxylated flavonols and dihydroflavonols (astilbin and engeletin, respectively mono- and dihydroxylated on the B-ring). The most abundant flavanol subunits (and also the sum of tannins) were grouped in cluster **b**, and linked with cluster **c** containing anthocyanin-flavanol derivatives linked with an ethyl-bridge. Cluster **d** grouped hydroxycinnamates and several of their reaction products with anthocyanins (pyranoanthocyanins and caftaric-anthocyanins). Most of the flavanol monomers and terminal units are clustered in the close **e1** and **e2**. Clusters **f1** and **f2** contained respectively mono-and di-hydroxylated anthocyanins along with some of their derivatives and trihydroxylated anthocyanins. The latter encompassed **g1** and **g2**, containing trihydroxylated flavonols. It is also noticeable that β-glucogallin was included in **f2**. All stilbenes shared the same response to irrigation and were clustered in cluster **h**.

**Figure 8:**
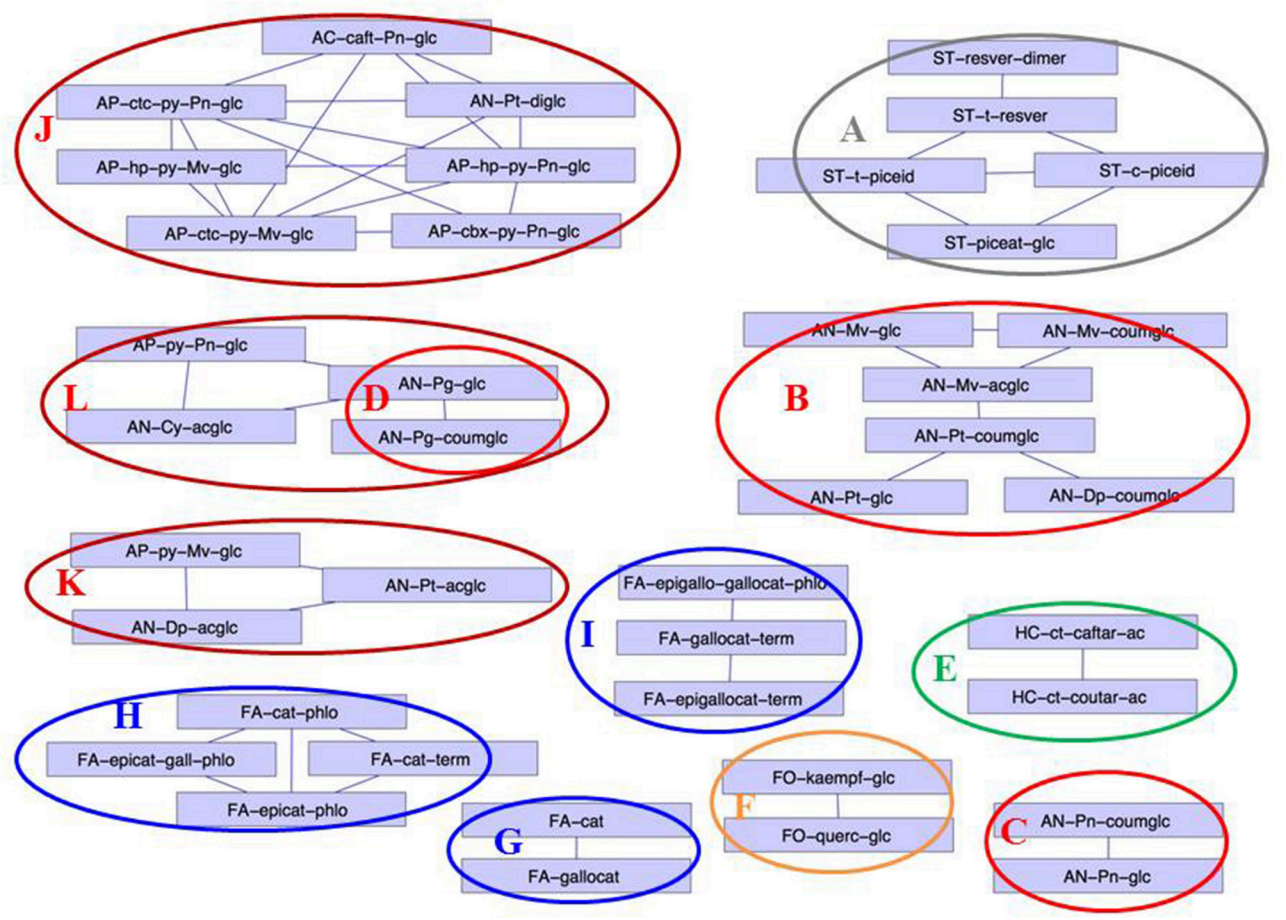
Correlation network (correlations >0.8) established from the Log (concentration I/concentration NI), calculated from the 105 MRM polyphenol composition variables (in mg berry^−1^, coded as in Table 1) on the 2014 data set. Clusters of the different polyphenol families are colored differently: anthocyanins (red), anthocyanin derived pigments (dark red), flavonols (yellow), flavan-3-ols (blue), stilbenes (gray), hydroxycinnamic acids (green).

The same data (log (irrigated/non-irrigated), calculated from polyphenol concentrations expressed in mg berry^−1^) was used to establish the correlation network shown in Figure 8. Only the correlations >0.8 are presented. Major clusters corresponded to stilbenes (**A**), native anthocyanins derived from delphinidin, petunidin, and malvidin (**B**), from peonidin (**C**), and from pelargonidin (**D**), caftaric and coutaric acids (**E**), kaempferol and quercetin 3-glucosides (**F**), catechin and gallocatechin monomers (**G**), (epi)catechin units of tannins (**H**), (epi)gallocatechin units of tannins (**I**), and anthocyanin derivatives, especially phenylpyranoanthocyanins and caftaric-anthocyanin adducts (**J**). Two additional clusters consisted of pyranopeonidin 3-glucoside, cyanidin 3-acetylglucoside and cluster D (**K**) and pyranomalvidin 3-glucoside with the 3-acetylglucosides of delphinidin and petunidin (**L**).

**Figure 9:**
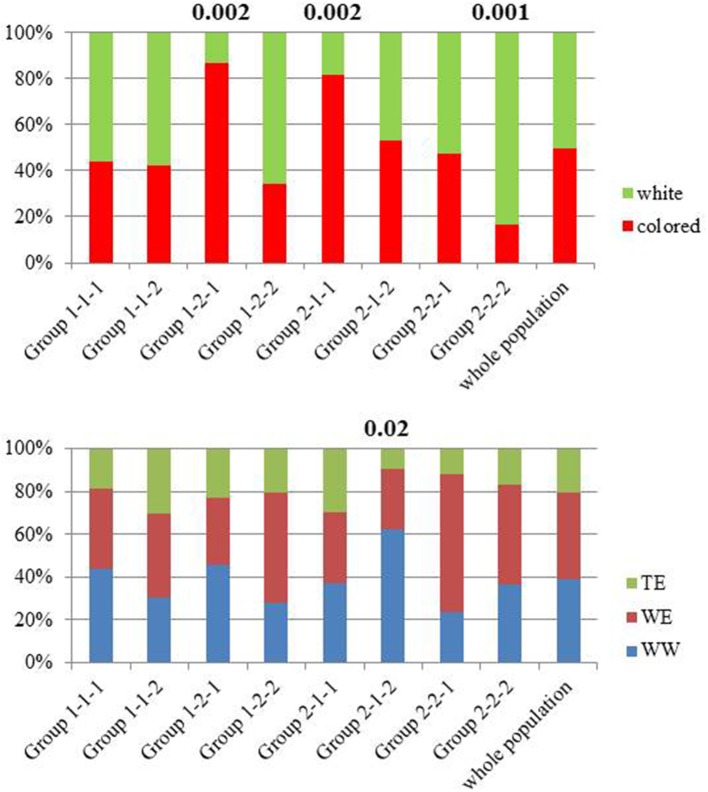
Histogram of the distribution in the eight cultivar subgroups arising from unsupervised hierarchical clustering of metabolites and cultivars affected by drought, calculated for all variables [Log (content I/content NI), with polyphenol concentrations expressed in mg berry^−1^] on the 2014 data set (Figure 7) and in the whole population of white and colored cultivars **(top)** and of wine West (WW), wine East (WE), and table East (TE) cultivars **(bottom)**. Significant differences between the distribution in a subgroup and that of the entire population are indicated by the corresponding Chi-2 values (*p* < 0.1) (cf. Table S3).

**Figure 10:**
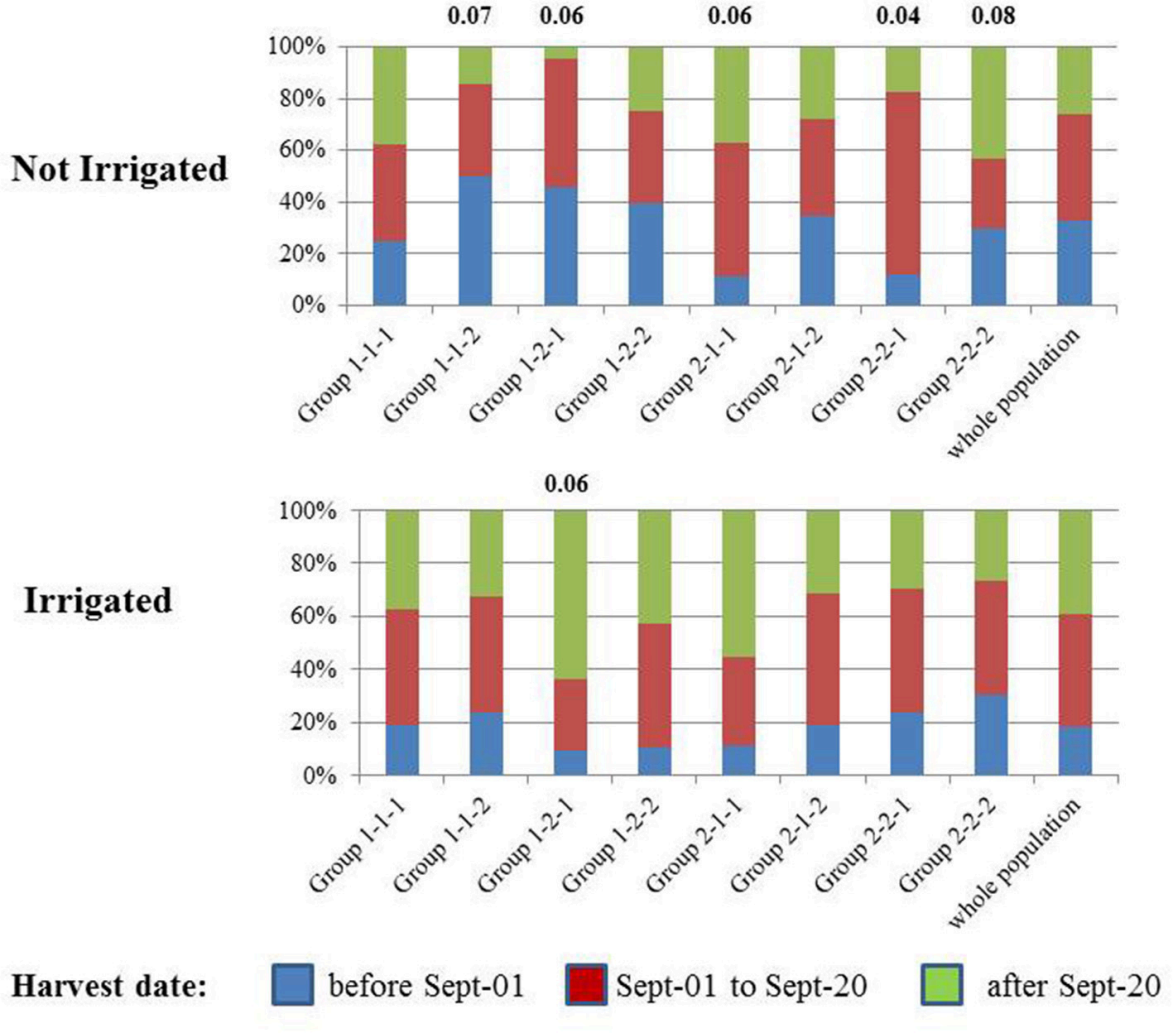
Histogram of the distribution of harvest dates in the eight cultivar subgroups arising from unsupervised hierarchical clustering of metabolites and cultivars affected by drought, calculated for all variables [Log (content I/content NI), with polyphenol concentrations expressed in mg berry^−1^] on the 2014 data set (Figure 7) and in the whole population in not irrigated **(Top)** and irrigated **(Bottom)** conditions. Significant differences between the distribution in a subgroup and that of the entire population are indicated by the corresponding Chi-2 values (*p* < 0.1) (cf. Table S3).

Berry anthocyanin, flavonol, hydroxycinnamic acid, and stilbene contents were increased or decreased under irrigated conditions in some cultivars. Groups of cultivars whose composition varies similarly in response to irrigation are clustered together (Figure 7). For example, irrigation resulted in increased and decreased stilbene levels in most cultivars of group 1 and group 2, respectively. The opposite pattern was observed for flavan-3-ols. The distribution of colored (i.e., black, red, and pink) cultivars and white cultivars, and that of the three genetic groups (WW, WE, TE) in some of the subgroups defined by unsupervised hierarchical clustering has been compared to that of the whole population (Figure 9). Chi2 tests performed on each subgroup showed that colored cultivars are overrepresented in subgroups 1-2-1 and 2-1-1 and underrepresented in subgroup 2-2-2 and cultivars from WW origin are overrepresented in subgroup 2-1-2 (Table S3). Although both contain mostly colored cultivars, subgroups 1-2-1 and 2-1-1 show different response to irrigation, with decreased anthocyanins (Figure 7, **f1,f2**), B-ring trihydroxylated flavonols (**g1, g2**) and stilbenes (**h**) in the latter, and reduced tannins (**b, e1, e2**) and increased stilbenes (**h**) in the former. Distributions of the harvest dates for each group under irrigated and not-irrigated conditions were also examined (Figure 10). Chi-2 tests (Table S3) performed on the entire population showed that the distribution of harvest dates was similar for all subgroups under irrigated conditions but significantly different under not irrigated conditions. Chi-2 test values calculated for each subgroup indicated that the distribution of harvest dates in some of them was significantly different from that of the whole population, although the difference was significant at *p* = 0.05 only for 2-2-1. Thus, not irrigated cultivars of subgroups 1-1-2 and 1-2-1 and cultivars of subgroups 2-1-1, 2-2-1, and 2-2-2 were harvested earlier and later, respectively, and cultivars of subgroup 1-2-1 were shifted toward later harvest dates under irrigated conditions.

## Discussion

### Cultivar differences in the polyphenol composition of grape berry skins

Major polyphenol families detected in berry skin samples were flavan-3-ols, including monomers and proanthocyanidins, anthocyanins, flavonols, hydroxycinnamic acids, and stilbenes, along with lower amounts of dihydroflavonols and benzoic acids, as classically reported. All families showed wide ranges of concentrations across the diversity panel. Anthocyanin contents enable distinction between white, pink, and red cultivars (Castellarin and Di Gaspero, 2007; Pelsy, 2010) although white grape berries also contain trace amounts of anthocyanin pigments (Arapitsas et al., 2015). Red cultivars were also characterized by the presence of flavonols with trihydroxylated B-rings, i.e., derived from myricetin, laricitrin, and syringetin glycosides, which are known to be specific of red cultivars (Mattivi et al., 2006). Cultivar differences in hydroxycinnamic acid contents have also been reported and related to differences in cultivar susceptibility to enzymatic browning (Cheynier et al., 1990). Genetic determinism of flavonols has been studied through QTL analysis (Malacarne et al., 2015), but this information is still lacking for stilbenes. However, their concentrations are also believed to highly depend on environmental factors as they are involved in plant defense against UV exposure and fungal attacks (Teixeira et al., 2013). It is noteworthy that some of the samples contained very high levels of flavonols and especially of quercetin and kaempferol derivatives compared to values reported earlier (Mattivi et al., 2006). This may be related to environmental conditions as berry concentration of quercetin glycosides have been shown to increase dramatically following sunlight exposure (Price et al., 1995; Spayd et al., 2002; Downey et al., 2004). Similarly, the lack of correlation between the flavan-3-ol contents of berries collected from a population of 141 grapevines cultivars over 2 successive years indicated that tannin accumulation is mostly driven by environmental factors rather than genetically determined (Huang et al., 2012b). In contrast, qualitative profiles within the different polyphenol groups are known to be cultivar characteristics. Thus, chemotaxonomic approaches based on grape anthocyanin profiles (Roggero et al., 1988; Mazza, 1995; Fournier-Level et al., 2009) or hydroxycinnamic acid profiles (Boursiquot et al., 1986) have been proposed. Skin flavan-3-ol composition also appeared highly conserved between years, meaning that it is mostly linked to genetic factors (Huang et al., 2012b). Our data, showing high correlations between years and between irrigation regimes for qualitative polyphenol variables and low correlations as well as strong vintage effect for quantitative variables (Table 2), confirm that the polyphenol profiles depend on cultivar while contents are affected by environmental factors, as reported in the above cited literature.

In addition to the expected native anthocyanins, several anthocyanin derivatives were detected. Among them, caftaric anthocyanin adducts were present only in trace amounts. As these adducts result from enzymatic oxidation catalyzed by grape polyphenoloxidase (Sarni-Manchado et al., 1997), this indicates that no enzymatic oxidation took place during sample preparation. Pyranoanthocyanins and carboxypyrano-anthocyanins resulting from reaction of anthocyanins respectively with acetaldehyde (Cheynier et al., 1997) and pyruvic acid (Fulcrand et al., 1998) have been reported in grape (Arapitsas et al., 2015). Anthocyanin dimers have also been isolated from grape skins (Vidal et al., 2004b). Strong correlations between the levels of malvidin-3-glucoside and peonidin 3-glucoside and those of vitisin B and pyranopeonidin 3-glucoside, respectively (Figure 1), substantiate the hypothesis that these compounds are formed *in vivo*. Moreover, the high level of vitisin B detected in some cultivars indicates that acetaldehyde is present in subcellular compartments in rather large amounts together with anthocyanins. Two major groups of tannin-anthocyanin reaction products were also detected. Flavanol-anthocyanin adducts resulting from cleavage of tannins followed by addition with anthocyanins have been detected in wine (Salas et al., 2004) and in various fruits including grapes (Gonzalez-Paramas et al., 2006). Flavanol-ethyl-anthocyanins resulting from condensation of anthocyanins and flavanols with acetaldehyde are well known to occur in wine (Timberlake and Bridle, 1976; Arapitsas et al., 2012) and have been detected in cranberry extracts (Tarascou et al., 2011). Reactions of (epi)catechin, anthocyanins, and acetaldehyde yield complex mixtures of products, including pyranoanthocyanin, (epi)catechin-ethyl-anthocyanin, and (epi)catechin-ethyl-(epi)catechin derivatives (Vallverdú-Queralt et al., 2017a,b). Molecules of the last group have been detected in wine (Cheynier et al., 1997) but this is the first report of their presence in grape. Flavanol-anthocyanins correlated with their anthocyanin precursors (Figures 1, **A** and **B**) while flavanol-ethyl-anthocyanins formed a specific cluster (Figure 1, **H**). Although these molecules could also form during sample preparation, the levels reported here and the relatively long reaction rates compared to the duration of our extraction procedure suggest that they were present *in planta*.

Other correlations networks established for the 2014 data only (not shown) showed additional relationships between malvidin-3-glucoside and anthocyanin dimers (malvidin 3-glucoside and malvidin 3-glucoside–peonidin 3-glucoside), and syringic acid that arises from degradation of malvidin (Furtado et al., 1993; Vallverdú-Queralt et al., 2016) and between peonidin 3-glucoside and vanillic acid. Formation of syringic and vanillic acids respectively from malvidin 3-glucoside–peonidin 3-glucoside can be promoted by light and heat exposure (Furtado et al., 1993).

Correlations between variables (Figure 1) can be interpreted in terms of biosynthetic pathways. Indeed, anthocyanin and flavonol variables clustered together according to their B-ring substitution (trihydroxylated/dihydroxylated) and/or acylation pattern. Clustering of those compounds according to their B-ring hydroxylation pattern is consistent with the ability of F3'H and F3'5'H to use both anthocyanin and flavonols as substrate (Bogs et al., 2006). Flavonols clustered in three correlation networks corresponding to B-ring trihydroxylated compounds (myricetin, laricitrin, and syringetin derivatives) and other (mono or dihydroxylated) flavonols and substitution by glucose or glucuronic acid. This probably reflects the high sugar specificity of the already described *Vitis* flavonol glycosyltransferases: when VvGT5 is quite exclusively a glucuronyl donor, VvGT6 catalyzes both flavonol glucosylation and galactosylation (Ono et al., 2010). Flavan-3-ols also clustered following their B-ring hydroxylation pattern, (epi)-catechin flavanol units, and (epi)gallocatechin units forming different groups. Strong correlations between terminal units and the corresponding monomers likely reflect the analytical method as monomers contribute to terminal units. Moreover, upper units, detected as the corresponding phloroglucinol adducts, were separated from terminal units, suggesting that both types of units have different precursors, as already suspected (Stafford et al., 1982; Huang et al., 2012a). Acetylated anthocyanin derivatives were correlated, regardless of the anthocyanin B-ring substitution while glucosylated, coumaroylated, and caffeoylated anthocyanins derived from malvidin, petunidin, and delphinidin (trihydroxylated) and from other anthocyanidins formed distinct groups. Although the already characterized acyltransferase Vv3AT is able to use both aliphatic and aromatic acyl-CoA as substrate (Rinaldo et al., 2015), this suggests that anthocyanin acylation with acetic acid and with hydroxycinnamic acids could involve alternative biosynthetic mechanisms (Bontpart et al., 2015).

Finally, correlations of anthocyanins with their derivatives and degradation products (Figure 1, clusters **A** and **B**), and clustering of molecules such as anthocyanin dimers (Figure 1, **F**), flavanol-ethyl anthocyanins (Figure 1, **H**) and hydroxyphenyl- and catechyl-pyranoanthocyanins, resulting from anthocyanin reactions with *p*-coumaric and caffeic acid (Figure 1, **G**) reflect their formation from the same precursors and/or through identical reaction mechanisms.

### Impact of water deficit on the polyphenol composition of grape berry skins

Not-irrigated vines suffered water stress in 2014 but not in 2015. Indeed, water stress classically induces a decrease in berry weight (Roby et al., 2004; Bucchetti et al., 2011). In 2015, irrigation had no significant impact on berry weight, which indicates that berries were not exposed to any sufficient water stress regime to induce phenotypic changes. Corroborating this hypothesis, in 2015, berry weights were higher and δ^13^C values were lower than those of berries from irrigated vines in 2014. Accordingly, none of the polyphenol variables showed significant differences between berries from irrigated and not irrigated vines (Table 3). In contrast, in 2014, water stress induced significant loss of berry weight as well as significant differences on the concentration of several polyphenols. This confirms that polyphenols are part of the chemical arsenal allowing adaptive response to abiotic stress, being protective molecules against oxidative damages by scavenging Reactive Oxygen Species (ROS) produced during stress (Rontein et al., 2002).

Not irrigated berries contained higher levels of most phenolic compounds when expressed in mg g^−1^ fresh berry weight (Table 3, Figure 5). This concentration effect can be attributed to reduced berry size under water stress, as observed earlier (Roby et al., 2004; Bucchetti et al., 2011). However, shifts between irrigated and non-irrigated samples on the PCA performed on the phenolic composition data expressed in mg per berry (Figure 6) showed that water status affected polyphenol biosynthesis. Water deficiency is known to impact berry development, decrease berry weight, and modulate accumulation of secondary metabolites including polyphenols (Kennedy et al., 2002; Roby et al., 2004). Data available on a limited number of genotypes suggest that the response to moderate water stress differs depending on the level of irrigation and/or water stress, on the berry development stage when water deficit occurs and on the cultivar (Ojeda et al., 2002; Teixeira et al., 2013). Thus, several studies have shown an increase in the accumulation of stilbenes, flavonols, and anthocyanins and enhanced transcription of genes involved in these pathways following moderate water deficiency while other studies failed to observe these effects or even observed a decrease. In Syrah, water deficit applied before or after veraison resulted in an increase of total anthocyanin contents and differences in the anthocyanin profiles (Ollé et al., 2011). Data on the effect of environment on tannin biosynthesis is still scarce: water deficiency in Cabernet Sauvignon (Kennedy et al., 2002; Castellarin et al., 2007) or in Syrah (Ollé et al., 2011), or thermic variation in Merlot (Cohen et al., 2012) did not affect tannin accumulation. In another study, a decrease or increase in tannin accumulation was reported in Syrah exposed respectively to early (between anthesis and veraison) or late (after veraison) water stress (Ojeda et al., 2002). However, tannin accumulation might also be related to biotic stress exposure (Dixon et al., 2005). As well, the significant increase of *cis*-resveratrol and piceatannol concentrations observed in 2015 under irrigated conditions may be due to plant response to increased fungal pressure as stilbenes are known to be involved in defense against fungi (Jeandet et al., 2002).

ANOVA performed on the 2014 samples (Table 3) showed that the levels of 16 MRM variables expressed per berry were significantly higher in irrigated berries. Tannins were the major family affected by irrigation, with both quantitative (increase of catechin and epicatechin, detected as monomers, and as terminal and upper units of tannin chains, and of total flavan-3-ol levels) and qualitative (decrease of % B-ring trihydroxylated units and mean DP) variations. A decrease of tannin DP in irrigated vines has been reported earlier (Ojeda et al., 2002) and the proportion of B-ring trihydroxylated units was reduced in shaded berries (Cortell and Kennedy, 2006). Other affected compounds included piceatannol, caffeic acid, and pigments resulting from reactions of anthocyanins with hydroxycinnamic acids, i.e., caftaric-anthocyanin adducts, phenylpyranoanthocyanins and catechyl-pyranoanthocyanins.

Several groups of variables were affected by irrigation in the same way and formed clusters on the correlation networks established from the response of MRM variables to irrigation (log; irrigated/non-irrigated of the concentrations expressed in mg berry^−1^, Figure 8). Thus, clustering of phenylpyranoanthocyanins, catechylpyranoanthocyanins, and caftaric-anthocyanin adducts indicated that they were simultaneously increased upon irrigation. Stilbenes formed another cluster, indicating that they were not only closely related, as shown by clustering of their concentrations (Figure 1, **L**) but also impacted in the same way by water deficit (Figures 7, **h**, 8, **A**). Transcription of genes involved in the biosynthesis of stilbene precursors has been shown to increase and decrease in response to water stress in Cabernet Sauvignon and Chardonnay, respectively (Deluc et al., 2011).

Other clusters grouped together members of the different flavonoid families (anthocyanins, flavan-3-ols, and flavonols) which were further sorted according to their B-ring substitution pattern. Thus, malvidin, delphinidin, and petunidin derivatives (trisubstituted on the B-ring) and peonidin derivatives (disubstituted on the B-ring) formed different groups (Figures 7, f1 and **f2**, 8, **B** and **C**). Water deficit has been shown to enhance expression of flavonoid 3′,5′-hydroxylase (F3′5′H), involved in B-ring trihydroxylation, relative to that of flavonoid 3′-hydroxylase (F3′H), involved in B-ring dihydroxylation, and, consequently, to increase the proportion of B-ring trihydroxylated anthocyanins (Castellarin et al., 2007). An increase of *O*-methyltransferase expression also correlated with accumulation of malvidin and peonidin derivatives (Castellarin et al., 2007). However, in another study, water deficit applied before and after veraison affected anthocyanin composition differently, enhancement of malvidin accumulation being observed only with post-veraison stress (Ollé et al., 2011). Among flavonols, drought responses of kaempferol and quercetin glucosides (respectively mono and dihydroxylated on the B-ring) were correlated (Figures 7, **a**, 8, **F**). Quercetin glycosides have been shown to accumulate following UV exposure of the berry (Price et al., 1995) and are believed to play a role in UV protection. Interestingly, expression of F3′5′H and biosynthesis of B-ring trihydroxylated anthocyanins were reduced in tissues protected from light exposure by shading of the berries or accumulation of phenolic compounds acting as UV screens in external tissues (Guan et al., 2014). The presence of β-glucogallin in the same response group to water status as trihydroxylated flavonols and anthocyanins is unexpected. This compound is suspected to be an intermediate in the flavanol galloylation pathway (Bontpart et al., 2015). However, glucose ester of hydroxybenzoic acid was already described as glucose donor for flavonoid glucosylation (Nishizaki et al., 2013). In the flavanol family, (epi)catechin based upper tannin units clustered with terminal catechin (Figure 8, **H**) and (epi)gallocatechin tannin units formed a different cluster (Figure 8, **I**), again indicating different responses of tannins based on trihydroxylated and dihydroxylated B-rings. However, catechin and gallocatechin monomers formed another group (Figure 8, **G**), suggesting that biosynthesis of flavan-3-ol monomers and of proanthocyanidins are differently regulated. The last two clusters (**L** and **K**) were similarly based on dihydroxylated and trihydroxylated anthocyanin B-rings, respectively. However, unexpected correlations of these molecules with pyranoanthocyanins derived from reaction of acetaldehyde with other anthocyanins require further investigation.

### Genetic diversity of grapevine response to drought shown by metabolomics

Grouping of polyphenols according to their drought response across the diversity panel provided confirmation of the results of variance analysis (e.g., for flavan-3-ols). Additional correlation networks between molecules that had not been detected in the global ANOVA treatment suggest different responses of the molecular clusters in different cultivars. Unsupervised hierarchical clustering of cultivars and metabolites (including MRM data and calculated variables) affected by drought (Figure 7) was performed to explore this hypothesis. Molecules clustered by family and, within some families, by B-ring substitution pattern, confirming the impact of irrigation on some specific branches of the biosynthetic pathway shown by the correlation networks. Clustering of cultivars confirmed cultivar differences in the molecular response to drought. Some of these differences may be related to differences in polyphenol metabolism as some cultivars accumulate specific polyphenol classes (e.g., colored vs. white cultivars). Three cultivar subgroups comprised an excess of colored or white cultivars, compared to the whole population (Figure 9). However, color did not fully explain the clustering based on polyphenol response to drought. Genetic groups did not appear as a major factor although cultivars from genetic group WW were overrepresented in subgroup 2-1-2. Moreover, cultivars from group 1 were generally harvested earlier under not irrigated conditions than those of group 2 (Figure 10). Differences in precocity may also induce different polyphenol response to irrigation as some compounds such as flavan-3-ols and hydroxycinnamic acids are biosynthesized at early development stages while anthocyanins and flavonols accumulate after veraison. Finally, some of the observed responses may be indirect responses, for instance due to differences in cultivar responses to other types of stresses, such as UV-stress since water regime also impacts canopy (for instance for flavonols), or biotic stress (for stilbenes).

This preliminary study is based on only two vintages, with no treatment replicate for individual cultivars. However, some cultivars with contrasted responses to water stress have been identified and could be used in future more detailed studies. In particular, it will be of interest to also characterize the plant physiological status, to determine if these contrasted behaviors are related to the near iso/anisohydric phenomenon, analyze their stability and explore potential interferences with phenological stages and environmental factors.

### Large scale metabolomics studies shedding light on polyphenol composition

The MRM method used in this study was targeted on a large number of polyphenolic compounds, including 96 molecules analyzed directly and 9 additional compounds released after acid-catalyzed depolymerization of proanthocyanidins in the presence of pholoroglucinol. Such experiments had never been performed at a large scale, in terms of number of targeted molecules and of number of studied cultivars. The large data collected made it possible to establish correlation networks that confirmed previous knowledge and provided new information on grape polyphenol metabolism. Conversely, the patterns established validate interpretation of mass spectrometry data for most of the compounds analyzed. However, a few compounds appeared as outliers in some of the clusters, raising questions on their attribution. For example, the signal attributed to petunidin-3,5-diglucoside clustered with pigments derived from reactions of anthocyanins with phenolic acids, suggesting confusion or contamination with another molecule of this group. This will be explored further, potentially leading to the discovery of new compounds. Similarly, clustering of β-glucogallin with B-ring trihydroxylated anthocyanins and flavonols is surprising. This may be related to a role of glucogallin in their biosynthesis, as proposed above. However, formation of β-glucogallin may also reflect degradation of delphinidin and/or myricetin, followed by glucosylation of the resulting gallic acid. In this case, other anthocyanins are expected to follow the same catabolic process. For example, degradation of malvidin or syringetin and of peonidin or isorhamnetin should similarly yield syringoyl- and vanilloyl-glucose which have not been included in the molecular targets of the MRM method. Detection of these new molecules would help validate this hypothesis.

## Conclusions

A large scale experiment involving cultivation of an association panel of 279 *V. vinifera* cultivars designed to represent the genetic and phenotypic variation encountered in cultivated grapevine and metabolomics analysis targeted to a large number of polyphenolic compounds (polyphenomics) was performed in 2014 and 2015. Chemometrics analysis of the data showed large differences in polyphenol composition related to genetic factors, environmental factors (i.e., water stress), and genetic x environment interactions. Correlation networks shed light on the relationships between the different polyphenol metabolites and related biosynthetic pathways. In addition, detailed polyphenomics analysis confirmed that polyphenol reactions described in wine take place in the berries. Finally, this paper reports the first large scale study demonstrating an influence of water stress on the different classes of polyphenols but also cultivar differences in the types and extents of drought responses, with different molecules affected either positively or negatively and different impacts on grape and wine quality. This work will be the foundation for identifying the genetic basis of the drought differential response of the cultivars in term of polyphenol composition, through Genome-Wide Association Study.

## Author contributions

LP and AV developed the MRM methods and performed the analyses, MR developed and applied the extraction protocols, EM and AVQ interpreted the mass spectrometry data, NS supervised the metabolomics analysis, JB and NT performed the chemometrics analysis, LL, JP, AA, NT, NS, and VC conceived the designed research and interpreted the results. All authors contributed to drafting and/or critical revision of the work and approved the manuscript.

### Conflict of interest statement

The authors declare that the research was conducted in the absence of any commercial or financial relationships that could be construed as a potential conflict of interest.

## Supplementary material

Table S1 List of cultivars collected in 2014 and/or 2015, with their codes, genetic groups, colors, and harvest dates under irrigated (I) and not irrigated (NI) conditions.
