# Supplementary material for: Cultivar Diversity of Grape Skin Polyphenol Composition and Changes in Response to Drought Investigated by LC-MS Based Metabolomics
Source: Front Plant Sci. 2017 Oct 27;8:1826. doi: 10.3389/fpls.2017.01826 (PMC5663694; doi:10.3389/fpls.2017.01826)
Supplement: Table S1 — List of cultivars collected in 2014 and/or 2015, with their codes, genetic groups, colors, and harvest dates under irrigated (I) and not irrigated (NI) conditions. [file Table1.DOCX]

| **Code** | **Cultivar name** | **Subgroup** | | | | **Color** | **Harvest date** | | | |  |
| --- | --- | --- | --- | --- | --- | --- | --- | --- | --- | --- | --- |
|  |  |  | |  | | | **2014, NI** | **2014, I** | **2015, NI** | **2015, I** | |
| 0Mtp1004 | San Lorenzo | | WW | | Black | | 6-Aug | 29-Aug | 17-Aug | 10-Sept | |
| 0Mtp1005 | Sao Mamede | | WW | | White | | 18-Aug | 22-Aug | 21-Aug | 09-Sept | |
| 0Mtp1033 | Sasca (Collection Ravaz) | | WE | | White-pink | | 25-Aug | 29-Aug | 07-Sept | 09-Oct | |
| 0Mtp1068 | Skiadopoulo | | WE | | White | | 02-Oct | 06-Oct | 09-Oct | 16-Oct | |
| 0Mtp1072 | Souzao faux | | WW | | Black | | 02-Oct | 19-Sept |  |  | |
| 0Mtp1073 | Starinky | | WE | | White | | 01-Oct | 01-Oct |  |  | |
| 0Mtp1129 | Totika | | WE | | Black | | 05-Sept | 23-Sept | 03-Sept | 15-Sept | |
| 0Mtp1154 | Urbanitraube noir (Collection Oberlin) | | WE | | Black | | 20-Aug | 7-Aug | 19-Aug | 21-Aug | |
| 0Mtp1156 | Urmi dinka | | WE | | White | | 18-Aug | 25-Aug | 15-Sept | 18-Sept | |
| 0Mtp1176 | Verdelho tinto | | WW | | Black | | 8-Aug | 15-Sept | 03-Sept | 19-Aug | |
| 0Mtp1195 | Magdeleine noire des Charentes | | WW | | Black | | 01-Sept | 19-Sept | 12-Oct | 14-Oct | |
| 0Mtp1213 | Vulpea faux (Collection Ravaz) | | WE | | Black | | 23-Sept | 10-Sept | 25-Sept | 31-Aug | |
| 0Mtp1218 | Wildbacher de Hongrie (Collection Ravaz) | | WW | | Black | |  |  | 12-Oct | 14-Oct | |
| 0Mtp1235 | Tzimliansky belyi | | WE | | White | | 4-Aug | 27-Aug | 10-Aug | 12-Aug | |
| 0Mtp1293 | Plant de Pedebernade 1 | | WW | | Black | | 01-Sept | 10-Oct |  |  | |
| 0Mtp1512 | Plant de Chaudefonds 53 (Fardeau) | | WW | | Black | | 15-Sept | 20-Aug |  |  | |
| 0Mtp1513 | Grain | | WW | | Black | | 06-Oct | 10-Sept |  |  | |
| 0Mtp1553 | Morenzi | | WE | | Red | | 10-Sept | 23-Sept |  |  | |
| 0Mtp1676 | B 40-97 (Ramming) | | TE | | White | | 10-Sept | 25-Aug | 3-Aug | 15-Sept | |
| 0Mtp171 | Cahours | | WW | | Black | | 01-Sept | 15-Sept | 07-Sept | 10-Sept | |
| 0Mtp1724 | Plant de Vic 98-N-3 (Collection Torres S.A.) | | WW | | Black | | 17-Sept | 15-Sept | 28-Sept | 10-Sept | |
| 0Mtp1733 | Fortunato | | TE | | White | | 19-Sept | 20-Aug | 21-Sept | 19-Aug | |
| 0Mtp1747 | Galotta | | WW | | Black | | 25-Aug | 4-Aug | 17-Aug | 14-Aug | |
| 0Mtp1758 | Canella | | WE | | Black | | 05-Sept | 03-Sept | 07-Sept | 19-Aug | |
| 0Mtp1761 | Caprugnone | | WE | | Black | | 25-Aug | 03-Sept | 28-Aug | 09-Sept | |
| 0Mtp220 | Chami abiad | | TE | | White | | 22-Aug | 18-Aug |  |  | |
| 0Mtp29 | Angoor Kalan | | TE | | White | | 25-Aug | 01-Sept |  |  | |
| 0Mtp300 | Dili kaftar | | TE | | Pink | | 18-Aug | 25-Aug |  |  | |
| 0Mtp313 | Domina = Geilweilerhof 4-25-7 | | WW | | Black | | 6-Aug | 29-Jul | 07-Sept | 25-Sept | |
| 0Mtp318 | Doppel -Augen | | TE | | White | | 03-Sept | 22-Aug |  |  | |
| 0Mtp36 | Arinto tinto (Collection Soares Franco) | | WW | | Black | | 01-Sept | 17-Sept | 21-Sept | 09-Oct | |
| 0Mtp406 | Mourvèdre Goulé | | WE | | Black | | 01-Sept | 20-Aug | 18-Sept | 15-Sept | |
| 0Mtp408 | Garbanega faux (Istituto San Michele) | | TE | | White | | 20-Aug | 15-Sept | 07-Sept | 28-Sept | |
| 0Mtp416 | Gharbi | | TE | | White | | 25-Aug | 29-Aug | 07-Sept | 30-Sept | |
| 0Mtp440 | Graeco | | TE | | White | |  |  | 17-Aug | 03-Sept | |
| 0Mtp469 | Hagnos Zöld | | WE | | White | | 16-Sept | 23-Sept | 15-Sept | 18-Sept | |
| 0Mtp500 | Plant de Ponteilla (Jaubert) | | WE | | White | | 01-Sept | 23-Sept | 03-Sept | 30-Sept | |
| 0Mtp537 | Grosse Mérille | | WW | | Black | | 01-Oct | 01-Sept | 07-Sept | 19-Aug | |
| 0Mtp561 | Öreg Kadarka | | WE | | Black | | 06-Oct | 23-Sept | 21-Sept | 12-Oct | |
| 0Mtp569 | Kara oglan faux | | WE | | White | | 12-Aug | 25-Aug | 31-Aug | 10-Sept | |
| 0Mtp581 | Khikhvi | | TE | | White | | 18-Aug | 22-Aug | 10-Aug | 21-Aug | |
| 0Mtp633 | Casetta | | WW | | Black | | 18-Aug | 8-Aug | 31-Aug | 03-Sept | |
| 0Mtp636 | Lameiro | | WW | | White | | 18-Aug | 18-Aug |  |  | |
| 0Mtp739 | Morenoa | | WW | | Black | | 27-Aug | 25-Aug | 07-Sept | 15-Sept | |
| 0Mtp775 | Muscate (Collection Ravaz) | | WE | | White-pink | | 18-Aug | 22 -Aug | 7-Aug | 17-Aug | |
| 0Mtp796 | Nero grosso | | TE | | Black | | 05-Sept | 22-Sept | 10-Sept | 03-Sept | |
| 0Mtp799 | Nevoeira | | WW | | Black | | 10-Sept | 16-Sept | 15-Sept | 15-Sept | |
| 0Mtp811 | Asma | | TE | | Black | | 17-Sept | 02-Oct |  |  | |
| 0Mtp835 | Osteiner | | WW | | White | | 01-Sept | 18-Aug | 7-Aug | 14-Aug | |
| 0Mtp89 | Belle Denise | | WW | | Black | | 22-Aug | 06-Oct |  | 21-Aug | |
| 0Mtp952 | Ramisco | | WW | | Black | | 20-Aug | 15-Sept | 07-Sept | 09-Sept | |
| 0Mtp961 | Ribote rose | | WE | | Pink | | 15-Sept | 16-Sept |  |  | |
| 0Mtp982 | Rosaky rose faux | | WE | | Pink | | 02-Oct | 24-Sept |  |  | |
| 0Mtp995 | Roussette basse de Seyssel | | WW | | White | | 12-Aug | 03-Sept | 17-Aug | 31-Aug | |
| 114Mtp4 | Pagadebiti | | WE | | White | | 22-Sept | 02-Oct | 07-Sept | 09-Oct | |
| 11Mtp3 | Piquepoul noir | | WW | | Black | | 20-Aug | 03-Sept | 12-Oct | 09-Sept | |
| 1217Mtp1 | Claverie Coulard | | WW | | White | | 01-Oct | 02-Oct | 07-Sept | 09-Sept | |
| 1218Mtp1 | Tavkveri | | TE | | Black | | 02-Oct | 24-Sept | 14-Oct | 14-Oct | |
| 1232Mtp2 | Gros Cabernet | | WW | | Black | | 20-Aug | 22-Aug | 31-Aug | 03-Sept | |
| 1237Mtp1 | Rossara | | WE | | Black | | 03-Sept | 22-Aug | 10-Sept | 07-Sept | |
| 1245Mtp1 | Freisa | | WW | | Black | | 01-Sept | 25-Aug | 19-Aug | 31-Aug | |
| 1247Mtp1 | Baresana = Korithi aspro | | TE | | White | | 25-Aug | 22-Sept |  |  | |
| 124Mtp1 | Riminèse | | WE | | White | | 12-Aug | 18-Aug |  |  | |
| 1258Mtp1 | Verdea | | WE | | White | | 12-Sept | 03-Sept |  |  | |
| 1277Mtp6 | Primitivo | | WE | | Black | | 05-Sept | 22-Aug |  |  | |
| 1284Mtp1 | Montepulciano | | WE | | Black | | 01-Sept | 03-Sept | 07-Sept | 09-Sept | |
| 1287Mtp2 | Lagrein | | WW | | Black | | 14-Aug | 18-Aug | 19-Aug | 19-Aug | |
| 129Mtp1 | Chatus | | WW | | Black | | 22-Aug | 25-Aug | 26-Aug | 28-Sept | |
| 1301Mtp1 | Verdiso | | WW | | White | | 02-Oct | 16-Sept | 03-Sept | 10-Sept | |
| 1303Mtp3 | Catarratto bianco | | WE | | White | | 22-Aug | 05-Sept | 10-Sept | 14-Oct | |
| 1307Mtp1 | Ansonica | | WE | | White | | 12-Sept | 22-Aug | 18-Sept | 09-Sept | |
| 1314Mtp1 | Perricone | | TE | | Black | | 01-Sept | 15-Sept | 18-Sept | 31-Aug | |
| 1338Mtp1 | Bellone | | WE | | White | | 14-Aug | 25-Aug | 12-Aug | 18-Sept | |
| 1354Mtp2 | Bonamico | | WE | | Black | | 18-Aug | 27-Aug | 21-Sept | 21-Sept | |
| 139Mtp1 | Dureza | | WW | | Black | | 02-Oct | 02-Oct | 12-Oct | 12-Oct | |
| 1483Mtp3 | Souzao faux | | WW | | Black | | 01-Sept | 15-Sept | 03-Sept | 26-Aug | |
| 1493Mtp1 | Touriga nacional | | WW | | Black | | 05-Sept | 14-Aug | 21-Aug | 28-Aug | |
| 1494Mtp1 | Verdelho tinto femelle | | WW | | Black | | 03-Sept | 08-Sept | 07-Sept | 15-Sept | |
| 1531Mtp3 | Carcajolo | | WW | | Black | | 05-Sept | 01-Oct | 21-Sept | 09-Sept | |
| 154Mtp1 | Joubertin | | WW | | Black | | 23-Sept | 03-Sept |  |  | |
| 1563Mtp1 | Ag isioum | | TE | | White | | 12-Aug | 22-Aug | 28-juil | 28-juil | |
| 1570Mtp1 | Lisztes feher | | WE | | White | | 25-Aug | 01-Oct | 12-Oct | 26-Aug | |
| 1576Mtp2 | Heunisch schwarz | | WW | | White | | 01-Oct | 02-Oct | 10-Sept | 09-Oct | |
| 1578Mtp2 | Kövidinka | | WE | | Pink | | 05-Sept | 03-Sept | 02-Oct | 09-Oct | |
| 157Mtp3 | Corbeau | | WW | | Black | | 12-Sept | 03-Sept | 07-Sept | 21-Sept | |
| 1583Mtp3 | Affenthaler | | WW | | Black | | 31-juil | 18-Aug | 07-Sept | 10-Aug | |
| 1620Mtp1 | Tantovina | | WE | | White | | 29-Aug | 23-Sept | 09-Oct | 12-Oct | |
| 1628Mtp3 | Dinka zöld | | WE | | White | | 27-Aug | 01-Oct | 03-Sept | 09-Sept | |
| 1629Mtp1 | Beregi rozsas | | WE | | Pink | | 20-Aug | 16-Sept | 19-Aug | 10-Sept | |
| 1631Mtp1 | Blank blauer | | WE | | Red | | 15-Sept | 23-Sept |  |  | |
| 1636Mtp1 | Rudezusa | | WE | | Black | | 25-Aug | 15-Sept | 10-Sept | 07-Sept | |
| 1644Mtp1 | Babica crna | | WE | | Black | | 27-Aug | 25-Aug | 21-Aug | 10-Sept | |
| 1653Mtp1 | Ruzevina (Jelaska) | | WE | | White | | 27-Aug | 01-Sept | 18-Sept | 26-Aug | |
| 1654Mtp1 | Posip bijeli | | WE | | White | | 25-Aug | 8-Aug | 07-Sept | 14-Oct | |
| 1662Mtp3 | Rosa menna di vacca | | WE | | Red | | 06-Oct | 06-Oct |  |  | |
| 1667Mtp3 | Negru virtos | | WE | | Black | | 08-Sept | 15-Sept | 21-Sept | 14-Oct | |
| 1673Mtp5 | Chaouch blanc | | TE | | White | | 14-Aug | 12-Sept | 15-Sept | 15-Sept | |
| 167Mtp12 | Altesse | | WW | | White | | 05-Sept | 20-Aug | 10-Sept | 19-Aug | |
| 1715Mtp1 | Kasoufi de la Bekaa | | TE | | White | | 20-Aug | 22-Aug | 21-Aug | 09-Sept | |
| 1744Mtp1 | Chahnani | | TE | | White | | 01-Sept | 05-Sept | 26-Aug | 09-Oct | |
| 176Mtp1 | Mondeuse blanche | | WW | | White | | 01-Sept | 03-Sept | 07-Sept | 23-Sept | |
| 1784Mtp1 | Kartsiotis | | WE | | Black | | 01-Sept | 16-Sept | 23-Sept | 21-Aug | |
| 1797Mtp1 | Fokiano | | WE | | Red | | 12-Aug | 4-Aug | 3-Aug | 21-Aug | |
| 1805Mtp1 | Araklinos | | WE | | Black | | 01-Sept | 06-Oct | 12-Oct | 09-Oct | |
| 1815Mtp1 | Tachtas | | TE | | White | | 19-Sept | 08-Sept |  |  | |
| 1827Mtp1 | Mission | | WE | | Black | | 01-Sept | 29-Aug | 28-Sept | 02-Oct | |
| 1837Mtp4 | Arvine | | WW | | White | | 8-Aug | 27-Aug | 17-Aug | 14-Aug | |
| 1844Mtp2 | Malvasia istriana | | WE | | White | | 01-Sept | 14-Aug | 17-Aug | 03-Sept | |
| 1888Mtp1 | Balbut bijeli | | WE | | White | | 02-Oct | 29-Aug | 23-Sept | 10-Sept | |
| 188Mtp1 | Mècle | | WW | | Black | | 14-Aug | 29-Aug |  |  | |
| 1893Mtp1 | Hadari | | TE | | White | | 05-Sept | 08-Sept |  |  | |
| 18Mtp8 | Carignan | | WE | | Black | | 25-Aug | 15-Sept | 07-Sept | 30-Sept | |
| 1972Mtp1 | Garrido macho | | TE | | White | | 08-Sept | 27-Aug | 07-Sept | 30-Sept | |
| 1Mtp3 | Rivairenc = Aspiran noir | | WW | | Black | | 15-Sept | 23-Sept | 07-Sept | 28-Sept | |
| 2003Mtp1 | Azizi el Jaïa | | TE | | White | | 25-Aug | 15-Sept | 15-Sept | 15-Sept | |
| 203Mtp1 | Saint-Laurent | | WW | | Black | | 31-juil | 6-Aug |  |  | |
| 2043Mtp1 | Bianco d'Alessano | | WE | | White | | 06-Oct | 29-Aug | 30-Sept | 07-Sept | |
| 2057Mtp1 | Impigno | | WE | | White | | 14-Aug | 25-Aug | 15-Sept | 21-Oct | |
| 2066Mtp1 | Humagne blanc | | WW | | White | | 18-Aug | 27-Aug | 07-Sept | 18-Sept | |
| 2070Mtp2 | Cacaboué | | WW | | White | | 31-juil | 12-Aug |  |  | |
| 2104Mtp1 | Bogazkere | | TE | | Black | |  |  | 18-Sept | 18-Sept | |
| 2107Mtp1 | Dimrit | | WE | | Black | | 12-Aug | 11-Aug | 17-Aug | 26-Aug | |
| 210Mtp1 | Gouais blanc | | WE | | White | | 02-Oct | 23-Sept | 30-Sept | 12-Oct | |
| 2112Mtp1 | Razdani | | TE | | Black | | 03-Sept | 22-Aug | 03-Sept | 03-Sept | |
| 2136Mtp2 | Galbena de Odobesti | | WE | | White | | 2-Aug | 12-Sept | 21-Sept | 15-Sept | |
| 2174Mtp1 | Pozsonyi feher | | WE | | White | | 01-Oct | 16-Sept | 10-Sept | 21-Sept | |
| 219Mtp2 | Arbane | | WW | | White | | 11-Aug | 13-Aug | 12-Aug | 19-Aug | |
| 2225Mtp5 | Barlinka | | TE | | Black | | 02-Oct | 02-Oct | 14-Oct | 14-Oct | |
| 2247Mtp2 | Feteasca regala | | WE | | White | | 7-Aug | 22-Aug | 12-Aug | 19-Aug | |
| 226Mtp7 | Gascon | | WW | | Black | | 20-Aug | 25-Aug |  |  | |
| 227Mtp1 | Roublot | | WW | | White | | 6-Aug | 22-Aug | 7-Aug | 21-Aug | |
| 2282Mtp2 | July Muscat | | TE | | White | | 22-Aug | 22-Aug | 12-Aug | 21-Aug | |
| 2287Mtp1 | Verdeca = Lagorthi | | WE | | White | | 01-Sept | 15-Sept | 10-Sept | 03-Sept | |
| 2298Mtp2 | Basicata | | WE | | White | | 06-Oct | 02-Oct | 16-Oct | 16-Oct | |
| 2304Mtp1 | Platyracho | | WE | | White | | 27-Aug | 03-Sept | 17-Aug | 03-Sept | |
| 2317Mtp1 | Staphidampelo | | WE | | Black | | 14-Aug | 25-Aug | 07-Sept | 28-Aug | |
| 2318Mtp1 | Strophyliatico | | WE | | White | | 08-Sept | 10-Sept | 21-Sept | 28-Sept | |
| 2327Mtp1 | Breider 5-6 | | WW | | Pink | |  |  | 5-Aug | 10-Aug | |
| 2342Mtp1 | Albaranzeuli bianco | | WE | | White | | 18-Aug | 05-Sept |  |  | |
| 2348Mtp1 | Cococciola | | WE | | White | | 12-Sept | 17-Sept | 15-Sept | 21-Sept | |
| 2349Mtp1 | Lambrusco Marani | | WW | | Black | | 29-Aug | 22-Aug |  |  | |
| 2371Mtp1 | Fumin | | WW | | Black | | 01-Sept | 29-Aug | 03-Sept | 18-Sept | |
| 2373Mtp1 | Greco bianco | | WE | | White | | 19-Sept | 06-Oct | 14-Oct | 15-Sept | |
| 2460Mtp2 | Negru mare | | TE | | Black | | 20-Aug | 02-Oct |  |  | |
| 2471Mtp1 | Tsitsa Kaprei | | TE | | White | | 01-Oct | 01-Oct | 14-Oct | 16-Oct | |
| 2472Mtp1 | Galbena uriasa | | WE | | White | | 01-Sept | 17-Sept | 12-Oct | 16-Oct | |
| 2500Mtp1 | Alexandroouli | | TE | | Black | | 31-juil | 4-Aug | 19-Aug | 28-Aug | |
| 2505Mtp1 | Assyl kara | | TE | | Black | |  |  | 15-Sept | 23-Sept | |
| 252Mtp1 | Poulsard | | WW | | Black | | 29-Aug | 20-Aug | 10-Aug | 26-Aug | |
| 2543Mtp1 | Maingonnat 3 L 1 | | WE | | White | | 17-Sept | 01-Sept | 10-Sept | 10-Sept | |
| 257Mtp16 | Savagnin blanc | | WW | | White | | 12-Aug | 18-Aug | 10-Aug | 21-Aug | |
| 261Mtp2 | Argant | | WE | | Black | | 14-Aug | 27-Aug |  |  | |
| 2621Mtp2 | Nieddera | | WE | | Black | | 01-Sept | 01-Oct |  |  | |
| 2635Mtp1 | Koz ouzioum | | TE | | White | |  |  | 25-Sept | 15-Sept | |
| 2655Mtp1 | Sapéré otskhanouri faux | | TE | | Black | |  |  | 17-Aug | 21-Aug | |
| 2657Mtp1 | Soïaki | | TE | | White | |  |  | 28-Sept | 16-Oct | |
| 2683Mtp1 | Sorok Let Oktiabria | | WE | | Black | | 8-Aug | 11-Aug | 10-Aug | 12-Aug | |
| 2694Mtp1 | Landroter = Cornalin du Valais | | WW | | Black | | 14-Aug | 18-Aug | 03-Sept | 19-Aug | |
| 26Mtp2 | Clairette | | WW | | White | | 02-Oct | 01-Oct | 07-Sept | 09-Oct | |
| 2708Mtp1 | Emerald seedless | | TE | | White | | 27-Aug | 01-Sept | 26-Aug | 07-Sept | |
| 2709Mtp1 | Opsimo Edessis | | TE | | White | | 15-Sept | 06-Oct | 09-Oct | 12-Oct | |
| 2713Mtp1 | Mireille | | WW | | White | | 28-juil | 29-juil |  |  | |
| 2725Mtp1 | Bacchus = Geilweilerhof 33-29-133 | | WW | | White | | 18-Aug | 14-Aug | 03-Sept | 15-Sept | |
| 2745Mtp1 | Heroldrebe = We S 130 | | WE | | Black | | 12-Aug | 05-Sept | 19-Aug | 19-Aug | |
| 2747Mtp1 | Arinarnoa | | WW | | Black | | 29-Aug | 29-Aug | 07-Sept | 28-Aug | |
| 2774Mtp1 | Sultanina de Bulgarie | | TE | | White | | 25-Aug | 29-Aug |  |  | |
| 2844Mtp1 | Lialmigui | | TE | | White | | 23-Sept | 03-Sept |  |  | |
| 284Mtp4 | Roter Veltliner | | WE | | Pink | | 22-Aug | 16-Sept | 19-Aug | 10-Aug | |
| 2856Mtp1 | Sourkhak biely | | TE | | White | | 28-juil | 31-juil |  |  | |
| 2874Mtp2 | Centennial seedless | | TE | | White | | 28-juil | 22-Aug | 28-juil | 15-Sept | |
| 2886Mtp1 | Completer | | WW | | White | | 20-Aug | 12-Aug | 12-Aug | 19-Aug | |
| 2890Mtp1 | Baserri | | WW | | White | | 14-Aug | 18-Aug | 12-Aug | 12-Aug | |
| 2892Mtp1 | Donzelinho branco | | WW | | White | | 08-Sept | 01-Sept | 15-Sept | 18-Sept | |
| 2893Mtp2 | Autumn seedless | | TE | | White | |  |  | 19-Aug | 21-Aug | |
| 2902Mtp1 | Gantziandan | | TE | | White | | 15-Sept | 25-Aug | 07-Sept | 21-Sept | |
| 2953Mtp1 | Labrusco | | WW | | Black | |  |  | 07-Sept | 31-Aug | |
| 2968Mtp1 | Mourtaou | | WW | | Black | |  |  | 17-Aug | 21-Aug | |
| 2991Mtp1 | Achlamiche | | TE | | White | | 12-Sept | 08-Sept | 07-Sept | 18-Sept | |
| 2995Mtp1 | Asswad Abou Khislé | | TE | | Black | | 29-Aug | 15-Sept | 15-Sept | 09-Sept | |
| 3000Mtp1 | Zakynthino | | WE | | White | | 01-Sept | 15-Sept | 21-Sept | 26-Aug | |
| 3009Mtp1 | Balsamina (Collection Oberlin) | | WW | | White | | 4-Aug | 31-juil | 09-Oct | 28-juil | |
| 3016Mtp1 | Massirart | | WW | | Black | | 03-Sept | 12-Sept |  |  | |
| 308Mtp9 | Muscat d'Alexandrie | | TE | | White | | 27-Aug | 25-Aug | 21-Aug | 09-Sept | |
| 328Mtp2 | Petit Verdot | | WW | | Black | | 10-Sept | 29-Aug | 26-Aug | 21-Aug | |
| 329Mtp1 | Gheméra | | WE | | Red | | 08-Sept | 01-Oct |  |  | |
| 332Mtp1 | Semillon | | WW | | White | | 12-Sept | 23-Sept | 26-Aug | 31-Aug | |
| 344Mtp2 | Blanc Auba | | WW | | White | | 22-Aug | 01-Sept | 21-Sept | 12-Oct | |
| 349Mtp1 | Penouille | | WW | | Black | | 05-Sept | 01-Sept | 14-Aug | 02-Oct | |
| 372Mtp1 | Gibert | | WW | | Black | | 01-Oct | 16-Sept | 12-Oct | 09-Oct | |
| 380Mtp1 | Baroque | | WW | | White | | 08-Sept | 02-Oct | 14-Oct | 09-Oct | |
| 403Mtp1 | Aouillat | | WW | | White | | 03-Sept | 03-Sept | 26-Aug | 10-Sept | |
| 411Mtp1 | Camaraou noir | | WW | | Black | | 12-Aug | 03-Sept | 21-Aug | 28-Aug | |
| 413Mtp1 | Courbu | | WW | | White | | 20-Aug | 08-Sept | 19-Aug | 10-Sept | |
| 424Mtp2 | Lauzet | | WW | | White | | 20-Aug | 14-Aug | 17-Aug | 21-Aug | |
| 433Mtp1 | Razachie rosie | | WE | | Red | | 03-Sept | 23-Sept | 12-Oct | 14-Oct | |
| 43Mtp1 | Olivette rose | | TE | | Red | | 06-Oct | 01-Oct |  |  | |
| 442Mtp2 | Négret pounjut | | WW | | Black | | 01-Sept | 25-Aug |  |  | |
| 443Mtp19 | Mauzac | | WW | | White | | 01-Oct | 19-Sept | 23-Sept | 15-Sept | |
| 446Mtp3 | Len de l'El | | WW | | White | | 18-Aug | 18-Aug | 23-Sept | 10-Sept | |
| 44Mtp1 | Alba imputotato | | WE | | White | | 14-Aug | 14-Aug | 28-Aug | 07-Sept | |
| 50Mtp1 | Alfrocheiro preto | | WW | | Black | | 18-Aug | 14-Aug | 21-Aug | 31-Aug | |
| 569Mtp1 | Moscato giallo | | WE | | White | | 02-Oct | 06-Oct |  |  | |
| 577Mtp1 | Moscato di Terracina | | WE | | White | | 01-Sept | 16-Sept | 21-Sept | 07-Sept | |
| 585Mtp62 | Chasselas | | WW | | White | | 08-Sept | 15-Sept |  |  | |
| 601Mtp1 | Aromriesling | | WW | | White | | 7-Aug | 14-Aug |  |  | |
| 613Mtp1 | Kaisermuskat | | WW | | White | | 7-Aug | 25-Aug |  |  | |
| 629Mtp1 | Darkaia noir | | TE | | Black | | 22-Sept | 01-Sept | 12-Oct | 28-Sept | |
| 632Mtp2 | Frankenthal rouge foncé | | TE | | Black | | 22-Sept | 25-Aug |  |  | |
| 635Mtp1 | Hunisa | | TE | | Red | | 06-Oct | 06-Oct | 14-Oct | 12-Oct | |
| 672Mtp2 | Molinera gorda | | TE | | Red | | 22-Sept | 12-Sept | 07-Sept | 26-Aug | |
| 725Mtp1 | Kolliniatiko | | WE | | Red | | 03-Sept | 02-Oct | 09-Oct | 12-Oct | |
| 727Mtp1 | Nehelescol | | TE | | White | |  |  | 09-Oct | 09-Oct | |
| 735Mtp1 | Dabouki | | TE | | White | | 10-Sept | 22-Sept | 16-Oct | 25-Sept | |
| 744Mtp1 | Kolontar | | WE | | White | | 05-Sept | 23-Sept | 02-Oct | 15-Sept | |
| 749Mtp2 | Coarna alba | | WE | | White | | 02-Oct | 06-Oct | 09-Oct | 14-Oct | |
| 74Mtp31 | Ugni blanc | | WE | | White | | 01-Oct | 03-Sept | 03-Sept | 21-Sept | |
| 789Mtp1 | Genk Uzum | | WE | | White | | 06-Oct | 06-Oct | 14-Oct | 14-Oct | |
| 80Mtp1 | Gros Bourgogne | | WE | | White | | 8-Aug | 14-Aug | 09-Oct | 10-Sept | |
| 830Mtp1 | Salicette (Collection Parc de la Tête d'Or) | | WW | | White | | 31-juil | 4-Aug |  |  | |
| 86Mtp2 | Tibouren | | WW | | Black | | 14-Aug | 13-Aug | 12-Aug | 21-Aug | |
| 975Mtp3 | Pardina = Pirovano 130 | | TE | | White | | 20-Aug | 25-Aug |  |  | |
| 9Mtp3 | Morrastel | | WW | | Black | | 22-Sept | 01-Oct | 21-Sept | 09-Sept | |
