# Supplementary material for: Cultivar Diversity of Grape Skin Polyphenol Composition and Changes in Response to Drought Investigated by LC-MS Based Metabolomics
Source: Front Plant Sci. 2017 Oct 27;8:1826. doi: 10.3389/fpls.2017.01826 (PMC5663694; doi:10.3389/fpls.2017.01826)
Supplement: Supplementary file 2 [file Table2.DOCX]

Supplementary Material

Cultivar diversity of grape skin polyphenol composition and changes in response to drought investigated by LC-MS based metabolomics

Lucie Pinasseau, Anna Vallverdú-Queralt, Arnaud Verbaere, Maryline Roque, Emmanuelle Meudec, Loïc Le Cunff, Jean-Pierre Péros, Agnès Ageorges, Nicolas Sommerer, Jean-Claude Boulet, Nancy Terrier, Véronique Cheynier^*^

*** Correspondence:** Corresponding Author: [veronique.cheynier@inra.fr](mailto:veronique.cheynier@inra.fr)

**Supplementary Table S2**: Results of the ANOVA performed on the data of irrigated (I) and not-irrigated (NI) vines available in both 2014 and 2015; polyphenol composition data in microgram per berry and microgram per g of berry; variable codes are provided in Table 1.

| **Variable Code** | **Groups*** | **Mean** | SNK** |  | **Groups*** | **Mean** | SNK** |
| --- | --- | --- | --- | --- | --- | --- | --- |
|  |  |  |  |  |  |  |  |
| **Polyphenolic composition** | | μg / berry |  |  |  | μg / g of berry |  |
| AN-Pg-glc | 2015_I | 0.90 | A |  | 2015_I | 0.39 | A |
|  | 2015_NI | 0.80 | A |  | 2014_NI | 0.38 | A |
|  | 2014_NI | 0.66 | A |  | 2015_NI | 0.33 | A |
|  | 2014_I | 0.60 | A |  | 2014_I | 0.25 | A |
| AN-Cy-glc | 2015_I | 79.65 | A |  | 2015_I | 34.98 | A |
|  | 2015_NI | 68.70 | A |  | 2014_NI | 34.43 | A |
|  | 2014_NI | 58.96 | A |  | 2015_NI | 27.28 | A |
|  | 2014_I | 56.71 | A |  | 2014_I | 24.27 | A |
| AN-Dp-glc | 2015_I | 29.66 | A |  | 2014_NI | 19.08 | A |
|  | 2015_NI | 28.51 | A |  | 2015_I | 14.77 | A |
|  | 2014_NI | 27.38 | A |  | 2015_NI | 14.36 | A |
|  | 2014_I | 24.17 | A |  | 2014_I | 12.67 | A |
| AN-Pt-glc | 2015_NI | 31.99 | A |  | 2014_NI | 19.76 | A |
|  | 2015_I | 30.89 | A |  | 2015_NI | 16.01 | A |
|  | 2014_NI | 28.64 | A |  | 2015_I | 15.45 | A |
|  | 2014_I | 26.48 | A |  | 2014_I | 13.62 | A |
| AN-Pn-glc | 2015_I | 68.58 | A |  | 2014_NI | 36.87 | A |
|  | 2015_NI | 58.89 | A |  | 2015_I | 31.69 | A |
|  | 2014_NI | 58.72 | A |  | 2015_NI | 26.67 | A |
|  | 2014_I | 57.88 | A |  | 2014_I | 26.41 | A |
| AN-Mv-glc | 2015_NI | 203.47 | A |  | 2014_NI | 132.54 | A |
|  | 2014_NI | 191.82 | A |  | 2015_NI | 99.98 | A |
|  | 2014_I | 184.93 | A |  | 2014_I | 90.51 | A |
|  | 2015_I | 180.14 | A |  | 2015_I | 87.11 | A |
| AN-Cy-diglc | 2015_I | 0.4 | A |  | 2015_I | 0.16 | A |
|  | 2015_NI | 0.31 | A |  | 2014_NI | 0.14 | A |
|  | 2014_NI | 0.25 | A |  | 2015_NI | 0.12 | A |
|  | 2014_I | 0.24 | A |  | 2014_I | 0.09 | A |
| AN-Dp-diglc | 2014_NI | 0.31 | A |  | 2014_NI | 0.19 | A |
|  | 2015_I | 0.26 | A |  | 2015_NI | 0.12 | A |
|  | 2015_NI | 0.25 | A |  | 2014_I | 0.12 | A |
|  | 2014_I | 0.24 | A |  | 2015_I | 0.11 | A |
| AN-Pt-diglc | 2015_I | 0.14 | A |  | 2015_I | 0.06 | A |
|  | 2015_NI | 0.13 | A |  | 2015_NI | 0.05 | A |
|  | 2014_I | 0.03 | B |  | 2014_NI | 0.01 | B |
|  | 2014_NI | 0.03 | B |  | 2014_I | 0.01 | B |
| AN-Pn-diglc | 2015_I | 0.16 | A |  | 2015_I | 0.06 | A |
|  | 2015_NI | 0.14 | A |  | 2015_NI | 0.06 | A |
|  | 2014_I | 0.05 | B |  | 2014_NI | 0.03 | B |
|  | 2014_NI | 0.05 | B |  | 2014_I | 0.02 | B |
| AN-Mv-diglc | 2015_NI | 0.22 | A |  | 2015_NI | 0.09 | A |
|  | 2015_I | 0.2 | A |  | 2015_I | 0.08 | A |
|  | 2014_NI | 0.11 | B |  | 2014_NI | 0.07 | A B |
|  | 2014_I | 0.1 | B |  | 2014_I | 0.04 | B |
| AN-Pg-acglc | 2015_I | 0.15 | A |  | 2015_I | 0.06 | A |
|  | 2015_NI | 0.14 | A |  | 2015_NI | 0.06 | A |
|  | 2014_I | 0.06 | B |  | 2014_NI | 0.03 | B |
|  | 2014_NI | 0.05 | B |  | 2014_I | 0.03 | B |
| AN-Cy-acglc | 2015_I | 3.47 | A |  | 2014_NI | 2.11 | A |
|  | 2014_I | 3.16 | A |  | 2015_I | 1.78 | A |
|  | 2014_NI | 2.99 | A |  | 2014_I | 1.77 | A |
|  | 2015_NI | 2.72 | A |  | 2015_NI | 1.31 | A |
| AN-Dp-acglc | 2015_I | 8.8 | A |  | 2014_NI | 6.09 | A |
|  | 2014_I | 8.55 | A |  | 2014_I | 5.05 | A |
|  | 2014_NI | 7.97 | A |  | 2015_I | 4.69 | A |
|  | 2015_NI | 7.75 | A |  | 2015_NI | 4.35 | A |
| AN-Pt-acglc | 2015_I | 8.85 | A |  | 2014_NI | 5.99 | A |
|  | 2014_I | 8.69 | A |  | 2014_I | 4.94 | A |
|  | 2015_NI | 8.44 | A |  | 2015_I | 4.78 | A |
|  | 2014_NI | 7.84 | A |  | 2015_NI | 4.59 | A |
| AN-Pn-acglc | 2015_I | 12.26 | A |  | 2014_NI | 6.62 | A |
|  | 2015_NI | 11.3 | A |  | 2015_I | 6.19 | A |
|  | 2014_NI | 9.61 | A |  | 2015_NI | 5.62 | A |
|  | 2014_I | 9.38 | A |  | 2014_I | 4.94 | A |
| AN-Mv-acglc | 2015_NI | 74.83 | A |  | 2014_NI | 43.19 | A |
|  | 2014_I | 66.73 | A |  | 2015_NI | 37.85 | A |
|  | 2015_I | 65.38 | A |  | 2014_I | 35.45 | A |
|  | 2014_NI | 57.9 | A |  | 2015_I | 33.88 | A |
| AN-Pg-coumglc | 2015_NI | 0.37 | A |  | 2015_NI | 0.16 | A |
|  | 2015_I | 0.35 | A |  | 2015_I | 0.15 | A |
|  | 2014_NI | 0.24 | A |  | 2014_NI | 0.15 | A |
|  | 2014_I | 0.24 | A |  | 2014_I | 0.11 | A |
| AN-Cy-coumglc | 2015_I | 17.3 | A |  | 2015_I | 7.67 | A |
|  | 2015_NI | 16.93 | A |  | 2015_NI | 7.11 | A |
|  | 2014_I | 11.32 | A |  | 2014_NI | 6.91 | A |
|  | 2014_NI | 11.19 | A |  | 2014_I | 5.41 | A |
| AN-Dp-coumglc | 2015_NI | 47.47 | A |  | 2014_NI | 23.85 | A |
|  | 2015_I | 41.91 | A |  | 2015_NI | 22.92 | A |
|  | 2014_NI | 33.58 | A |  | 2015_I | 20.66 | A |
|  | 2014_I | 32.89 | A |  | 2014_I | 17.25 | A |
| AN-Pt-coumglc | 2015_NI | 39.98 | A |  | 2014_NI | 19.91 | A |
|  | 2015_I | 32.56 | A |  | 2015_NI | 18.82 | A |
|  | 2014_NI | 27.65 | A |  | 2015_I | 15.68 | A |
|  | 2014_I | 27.64 | A |  | 2014_I | 14.1 | A |
| AN-Pn-coumglc | 2015_NI | 46.8 | A |  | 2015_I | 21.2 | A |
|  | 2015_I | 44.66 | A |  | 2015_NI | 20.2 | A |
|  | 2014_I | 31.22 | A |  | 2014_NI | 19.35 | A |
|  | 2014_NI | 30.41 | A |  | 2014_I | 134.48 | A |
| AN-Mv-coumglc | 2015_NI | 286.14 | A |  | 2015_NI | 130.37 | A |
|  | 2015_I | 205.58 | A |  | 2014_NI | 123.76 | A |
|  | 2014_NI | 181.71 | A |  | 2015_I | 95.38 | A |
|  | 2014_I | 172.62 | A |  | 2014_I | 85.33 | A |
| AN-Cy-caffglc | 2015_I | 0.22 | A |  | 2015_I | 0.09 | A |
|  | 2015_NI | 0.18 | A |  | 2015_NI | 0.07 | A B |
|  | 2014_I | 0.1 | B |  | 2014_NI | 0.05 | B C |
|  | 2014_NI | 0.09 | B |  | 2014_I | 0.04 | C |
| AN-Dp-caffglc | 2015_I | 0.21 | A |  | 2015_I | 0.09 | A |
|  | 2015_NI | 0.2 | A |  | 2015_NI | 0.08 | A |
|  | 2014_I | 0.1 | B |  | 2014_NI | 0.05 | A |
|  | 2014_NI | 0.08 | B |  | 2014_I | 0.05 | A |
| AN-Pt-caffglc | 2015_NI | 0.2 | A |  | 2015_NI | 0.08 | A |
|  | 2015_I | 0.2 | A |  | 2015_I | 0.08 | A |
|  | 2014_I | 0.12 | B |  | 2014_NI | 0.07 | A |
|  | 2014_NI | 0.11 | B |  | 2014_I | 0.06 | A |
| AN-Pn-caffglc | 2015_I | 0.61 | A |  | 2015_I | 0.28 | A |
|  | 2015_NI | 0.5 | A B |  | 2014_NI | 0.23 | A |
|  | 2014_I | 0.38 | B |  | 2015_NI | 0.22 | A |
|  | 2014_NI | 0.33 | B |  | 2014_I | 0.18 | A |
| AN-Mv-caffglc | 2015_NI | 1.69 | A |  | 2015_NI | 0.79 | A |
|  | 2015_I | 1.53 | A |  | 2015_I | 0.71 | A |
|  | 2014_I | 1.13 | A |  | 2014_NI | 0.67 | A |
|  | 2014_NI | 0.93 | A |  | 2014_I | 0.53 | A |
| AN-Mv-glc-Pn-glc | 2015_I | 0.15 | A |  | 2015_I | 0.06 | A |
|  | 2015_NI | 0.14 | A |  | 2015_NI | 0.06 | A |
|  | 2014_I | 0.08 | B |  | 2014_NI | 0.04 | B |
|  | 2014_NI | 0.07 | B |  | 2014_I | 0.04 | B |
| AN-Mv-glc-dimer | 2015_I | 0.15 | A |  | 2015_I | 0.06 | A |
|  | 2015_NI | 0.14 | A |  | 2015_NI | 0.06 | A |
|  | 2014_I | 0.08 | B |  | 2014_NI | 0.04 | B |
|  | 2014_NI | 0.07 | B |  | 2014_I | 0.03 | B |
| AP-py-Pn-glc | 2015_I | 4.18 | A |  | 2014_NI | 2.22 | A |
|  | 2015_NI | 3.36 | A |  | 2015_I | 2.02 | A |
|  | 2014_NI | 3.32 | A |  | 2015_NI | 1.48 | A |
|  | 2014_I | 2.47 | A |  | 2014_I | 1.17 | A |
| AP-py-Mv-glc | 2015_NI | 18.3 | A |  | 2014_NI | 9.98 | A |
|  | 2015_I | 16.48 | A |  | 2015_NI | 9.06 | A |
|  | 2014_NI | 14.2 | A |  | 2015_I | 8.39 | A |
|  | 2014_I | 10.91 | A |  | 2014_I | 5.55 | A |
| AP-hp-py-Pn-glc | 2015_I | 0.14 | A |  | 2015_I | 0.06 | A |
|  | 2015_NI | 0.13 | A |  | 2015_NI | 0.05 | A |
|  | 2014_I | 0.03 | B |  | 2014_NI | 0.01 | B |
|  | 2014_NI | 0.03 | B |  | 2014_I | 0.01 | B |
| AP-hp-py-Mv-glc | 2015_I | 0.14 | A |  | 2015_I | 0.06 | A |
|  | 2015_NI | 0.13 | A |  | 2015_NI | 0.05 | A |
|  | 2014_I | 0.04 | B |  | 2014_NI | 0.02 | B |
|  | 2014_NI | 0.03 | B |  | 2014_I | 0.01 | B |
| AP-ctc-py-Pn-glc | 2015_I | 0.11 | A |  | 2015_I | 0.04 | A |
|  | 2015_NI | 0.1 | A |  | 2015_NI | 0.04 | A |
|  | 2014_I | 0.04 | B |  | 2014_NI | 0.01 | B |
|  | 2014_NI | 0.03 | B |  | 2014_I | 0.01 | B |
| AP-ctc-py-Mv-glc | 2015_I | 0.11 | A |  | 2015_I | 0.04 | A |
|  | 2015_NI | 0.1 | A |  | 2015_NI | 0.04 | A |
|  | 2014_I | 0.03 | B |  | 2014_NI | 0.01 | B |
|  | 2014_NI | 0.03 | B |  | 2014_I | 0.01 | B |
| AP-cbx-py-Pn-glc | 2015_I | 0.15 | A |  | 2015_I | 0.06 | A |
|  | 2015_NI | 0.14 | A |  | 2015_NI | 0.05 | A |
|  | 2014_I | 0.04 | B |  | 2014_NI | 0.02 | B |
|  | 2014_NI | 0.03 | B |  | 2014_I | 0.01 | B |
| AP-cbx-py-Mv-glc | 2015_NI | 0.4 | A |  | 2014_NI | 0.2 | A |
|  | 2015_I | 0.37 | A |  | 2015_NI | 0.18 | A |
|  | 2014_I | 0.34 | A |  | 2015_I | 0.16 | A |
|  | 2014_NI | 0.3 | A |  | 2014_I | 0.15 | A |
| AF-Pt-glc-(epi)cat | 2015_I | 0.15 | A |  | 2015_I | 0.06 | A |
|  | 2015_NI | 0.13 | A |  | 2015_NI | 0.05 | A B |
|  | 2014_I | 0.08 | B |  | 2014_NI | 0.04 | B C |
|  | 2014_NI | 0.06 | B |  | 2014_I | 0.04 | C |
| AF-Pn-glc-(epi)cat | 2015_I | 0.2 | A |  | 2014_NI | 0.11 | A |
|  | 2015_NI | 0.18 | A |  | 2015_I | 0.09 | A |
|  | 2014_I | 0.16 | A |  | 2015_NI | 0.08 | A |
|  | 2014_NI | 0.16 | A |  | 2014_I | 0.08 | A |
| AF-Mv-glc-(epi)gallocat | 2015_I | 0.15 | A |  | 2015_I | 0.06 | A |
|  | 2015_NI | 0.14 | A B |  | 2015_NI | 0.05 | A |
|  | 2014_NI | 0.09 | B C |  | 2014_NI | 0.05 | A |
|  | 2014_I | 0.07 | C |  | 2014_I | 0.03 | A |
| AF-Mv-glc-(epi)cat | 2015_I | 0.16 | A |  | 2015_I | 0.06 | A |
|  | 2015_NI | 0.13 | A B |  | 2015_NI | 0.05 | A |
|  | 2014_I | 0.1 | B C |  | 2014_I | 0.04 | A |
|  | 2014_NI | 0.06 | C |  | 2014_NI | 0.04 | A |
| AF-(epi)gallocat-Pn-glc | 2015_NI | 0.48 | A |  | 2015_NI | 0.23 | A |
|  | 2015_I | 0.46 | A |  | 2015_I | 0.22 | A |
|  | 2014_NI | 0.15 | B |  | 2014_NI | 0.09 | B |
|  | 2014_I | 0.13 | B |  | 2014_I | 0.06 | B |
| AF-(epi)gallocat-Mv-glc | 2015_NI | 0.48 | A |  | 2015_NI | 0.23 | A |
|  | 2015_I | 0.42 | A B |  | 2015_I | 0.19 | A |
|  | 2014_NI | 0.28 | B |  | 2014_NI | 0.19 | A |
|  | 2014_I | 0.25 | B |  | 2014_I | 0.12 | A |
| AF-(epi)cat-Pn-glc | 2015_I | 0.32 | A |  | 2015_I | 0.14 | A |
|  | 2015_NI | 0.29 | A |  | 2015_NI | 0.12 | A |
|  | 2014_I | 0.11 | B |  | 2014_NI | 0.05 | B |
|  | 2014_NI | 0.1 | B |  | 2014_I | 0.04 | B |
| AF-(epi)cat-Mv-glc | 2015_NI | 0.62 | A |  | 2015_NI | 0.29 | A |
|  | 2015_I | 0.52 | A |  | 2015_I | 0.24 | A |
|  | 2014_NI | 0.18 | B |  | 2014_NI | 0.11 | B |
|  | 2014_I | 0.16 | B |  | 2014_I | 0.07 | B |
| AF-(epi)cat-eth-Pn-glc-i1 | 2015_I | 0.15 | A |  | 2015_I | 0.06 | A |
|  | 2015_NI | 0.14 | A |  | 2015_NI | 0.05 | A |
|  | 2014_I | 0.04 | B |  | 2014_NI | 0.02 | B |
|  | 2014_NI | 0.38 | B |  | 2014_I | 0.02 | B |
| AF-(epi)cat-eth-Pn-glc-i2 | 2015_I | 0.17 | A |  | 2015_I | 0.08 | A |
|  | 2015_NI | 0.13 | A B |  | 2015_NI | 0.05 | A B |
|  | 2014_I | 0.1 | A B |  | 2014_I | 0.04 | A B |
|  | 2014_NI | 0.05 | B |  | 2014_NI | 0.03 | B |
| AF-(epi)cat-eth-Pn-glc-i3 | 2014_I | 0.29 | A |  | 2014_I | 0.11 | A |
|  | 2015_I | 0.22 | A |  | 2015_I | 0.09 | A |
|  | 2015_NI | 0.18 | A |  | 2015_NI | 0.07 | A |
|  | 2014_NI | 0.07 | A |  | 2014_NI | 0.04 | A |
| AF-(epi)cat-eth-Pn-glc-i4 | 2015_I | 0.15 | A |  | 2015_I | 0.06 | A |
|  | 2015_NI | 0.14 | A |  | 2015_NI | 0.05 | A |
|  | 2014_I | 0.08 | B |  | 2014_I | 0.03 | B |
|  | 2014_NI | 0.05 | B |  | 2014_NI | 0.02 | B |
| AF-(epi)cat-eth-Mv-glc-i1 | 2015_NI | 0.18 | A |  | 2015_NI | 0.07 | A |
|  | 2015_I | 0.17 | A |  | 2015_I | 0.07 | A |
|  | 2014_I | 0.08 | B |  | 2014_I | 0.04 | A B |
|  | 2014_NI | 0.05 | B |  | 2014_NI | 0.03 | B |
| AF-(epi)cat-eth-Mv-glc-i2 | 2015_I | 0.31 | A |  | 2015_I | 0.12 | A |
|  | 2015_NI | 0.28 | A |  | 2015_NI | 0.11 | A |
|  | 2014_I | 0.14 | A |  | 2014_I | 0.06 | A |
|  | 2014_NI | 0.07 | A |  | 2014_NI | 0.04 | A |
| AF-(epi)cat-eth-Mv-glc-i3+4 | 2015_NI | 0.72 | A |  | 2015_NI | 0.3 | A |
|  | 2014_I | 0.55 | A |  | 2014_I | 0.26 | A |
|  | 2015_I | 0.45 | A |  | 2015_I | 0.18 | A |
|  | 2014_NI | 0.19 | A |  | 2014_NI | 0.13 | A |
| AC-caft-Pn-glc | 2015_NI | 0.15 | A |  | 2015_NI | 0.06 | A |
|  | 2015_I | 0.14 | A |  | 2015_I | 0.06 | A |
|  | 2014_I | 0.04 | B |  | 2014_NI | 0.01 | B |
|  | 2014_NI | 0.03 | B |  | 2014_I | 0.01 | B |
| AC-caft-Mv-glc | 2015_I | 0.14 | A |  | 2015_I | 0.06 | A |
|  | 2015_NI | 0.14 | A |  | 2015_NI | 0.05 | A |
|  | 2014_I | 0.04 | B |  | 2014_NI | 0.02 | B |
|  | 2014_NI | 0.03 | B |  | 2014_I | 0.01 | B |
| HF-taxif | 2015_NI | 1.91 | A |  | 2015_NI | 0.78 | A |
|  | 2015_I | 1.83 | A |  | 2015_I | 0.77 | A |
|  | 2014_I | 0.88 | B |  | 2014_NI | 0.4 | B |
|  | 2014_NI | 0.71 | B |  | 2014_I | 0.35 | B |
| HF-astilb | 2015_NI | 4.87 | A |  | 2015_NI | 1.8 | A |
|  | 2015_I | 4.79 | A |  | 2014_I | 1.73 | A |
|  | 2014_I | 3.92 | A |  | 2015_I | 1.68 | A |
|  | 2014_NI | 2.76 | A |  | 2014_NI | 1.57 | A |
| FO-syring-glucur | 2015_I | 0.09 | A |  | 2015_I | 0.03 | A |
|  | 2015_NI | 0.08 | A |  | 2015_NI | 0.03 | A |
|  | 2014_I | 0.04 | B |  | 2014_NI | 0.02 | B |
|  | 2014_NI | 0.03 | B |  | 2014_I | 0.01 | B |
| FO-syring-glc | 2015_NI | 10.88 | A |  | 2014_NI | 5.45 | A |
|  | 2014_I | 9,28 | A |  | 2015_NI | 4.94 | A |
|  | 2014_NI | 8,06 | A |  | 2014_I | 4.45 | A |
|  | 2015_I | 7,41 | A |  | 2015_I | 3.22 | A |
| FO-querc-glucur | 2015_I | 100.05 | A |  | 2014_NI | 43.41 | A |
|  | 2015_NI | 94,56 | A |  | 2015_I | 41.51 | A |
|  | 2014_I | 76,73 | B |  | 2015_NI | 38.45 | A |
|  | 2014_NI | 74,43 | B |  | 2014_I | 31.25 | B |
| FO-querc-glc | 2015_NI | 332.49 | A |  | 2015_I | 126.69 | A |
|  | 2015_I | 324.6 | A |  | 2014_NI | 123.25 | A |
|  | 2014_I | 222.05 | B |  | 2015_NI | 122.2 | A |
|  | 2014_NI | 219.77 | B |  | 2015_I | 88.73 | B |
| FO-myric-glucur | 2014_NI | 0.31 | A |  | 2014_NI | 0.2 | A |
|  | 2015_NI | 0.3 | A |  | 2015_NI | 0.13 | B |
|  | 2014_I | 0.28 | A |  | 2014_I | 0.12 | B |
|  | 2015_I | 0.26 | A |  | 2015_I | 0.11 | B |
| FO-myric-glc | 2015_NI | 19.32 | A |  | 2015_NI | 8.7 | A |
|  | 2015_I | 16.53 | A |  | 2014_NI | 8.04 | A |
|  | 2014_I | 12.52 | A |  | 2015_I | 7.32 | A |
|  | 2014_NI | 12.19 | A |  | 2014_I | 6 | A |
| FO-laric-glucur | 2015_NI | 0.09 | A |  | 2015_NI | 0.04 | A |
|  | 2015_I | 0.09 | A |  | 2015_I | 0.04 | A |
|  | 2014_I | 0.05 | B |  | 2014_NI | 0.03 | A |
|  | 2014_NI | 0.05 | B |  | 2014_I | 0.02 | B |
| FO-laric-glc | 2015_NI | 10,45 | A |  | 2014_NI | 5.84 | A |
|  | 2014_I | 9.81 | A |  | 2014_I | 4.77 | A |
|  | 2014_NI | 8.64 | A |  | 2015_NI | 4.74 | A |
|  | 2015_I | 7.9 | A |  | 2015_I | 3.46 | A |
| FO-kaempf-glucur | 2014_I | 0.15 | A |  | 2014_NI | 0.08 | A |
|  | 2014_NI | 0.15 | A |  | 2014_I | 0.05 | B |
|  | 2015_NI | 0.12 | A |  | 2015_NI | 0.05 | B |
|  | 2015_I | 0.12 | A |  | 2015_I | 0.05 | B |
| FO-kaempf-glc | 2015_NI | 220.78 | A |  | 2014_NI | 91.67 | A |
|  | 2015_I | 204.71 | A |  | 2015_NI | 77.01 | A |
|  | 2014_I | 178.37 | A |  | 2015_I | 75.19 | A |
|  | 2014_NI | 165.61 | A |  | 2014_I | 71.72 | A |
| FO-isorham-glucur | 2015_NI | 0.12 | A |  | 2014_NI | 0.07 | A |
|  | 2014_NI | 0.11 | A |  | 2015_NI | 0.05 | B |
|  | 2015_I | 0.11 | A |  | 2014_I | 0.05 | B |
|  | 2014_I | 0.11 | A |  | 2015_I | 0.04 | B |
| FO-isorham-glc | 2014_NI | 18.75 | A |  | 2014_NI | 11.42 | A |
|  | 2014_I | 17.87 | A |  | 2014_I | 8.12 | B |
|  | 2015_NI | 17.77 | A |  | 2015_NI | 7.4 | B |
|  | 2015_I | 15.73 | A |  | 2015_I | 6.33 | B |
| ST-c-resver | 2015_I | 0.22 | A |  | 2014_NI | 0.11 | A |
|  | 2014_NI | 0.2 | A |  | 2015_I | 0.1 | A |
|  | 2014_I | 0.2 | A |  | 2014_I | 0.08 | A B |
|  | 2015_NI | 0.18 | A |  | 2015_NI | 0.07 | B |
| ST-t-resver | 2015_I | 8.35 | A |  | 2015_I | 3.55 | A |
|  | 2015_NI | 6.86 | A |  | 2014_NI | 3.21 | A |
|  | 2014_I | 5.74 | A |  | 2015_NI | 2.86 | A |
|  | 2014_NI | 5.44 | A |  | 2014_I | 2.36 | A |
| ST-c-piceid | 2015_NI | 24.73 | A |  | 2014_NI | 15.02 | A |
|  | 2015_I | 24.65 | A |  | 2015_NI | 10.66 | A |
|  | 2014_NI | 23.98 | A |  | 2015_I | 10.55 | A |
|  | 2014_I | 23.95 | A |  | 2014_I | 9.41 | A |
| ST-t-piceid | 2015_NI | 8.16 | A |  | 2014_NI | 3.54 | A |
|  | 2015_I | 7.97 | A |  | 2015_NI | 3.39 | A |
|  | 2014_I | 6.58 | A |  | 2015_I | 3.21 | A |
|  | 2014_NI | 5.8 | A |  | 2014_I | 32.66 | A |
| ST-piceat-glc | 2015_I | 1.12 | A |  | 2015_I | 0.47 | A |
|  | 2015_NI | 1.05 | A |  | 2014_NI | 0.46 | A |
|  | 2014_I | 0.94 | A |  | 2015_NI | 0.45 | A |
|  | 2014_NI | 0.7 | A |  | 2014_I | 0.36 | A |
| ST-piceat | 2015_I | 0.53 | A |  | 2015_I | 0.25 | A |
|  | 2015_NI | 0.44 | A |  | 2015_NI | 0.19 | B |
|  | 2014_I | 0.32 | B |  | 2014_I | 0.13 | B |
|  | 2014_NI | 0.22 | B |  | 2014_I | 0.13 | B |
| ST-resver-dimer | 2015_NI | 1.3 | A |  | 2015_NI | 90.56 | A |
|  | 2015_I | 1.25 | A |  | 2015_I | 0.55 | A |
|  | 2014_I | 1.11 | A |  | 2014_NI | 0.49 | A |
|  | 2014_NI | 0.81 | A |  | 2014_I | 0.45 | A |
| FA-gallocat | 2014_I | 3.74 | A |  | 2014_NI | 1.87 | A |
|  | 2014_NI | 2.97 | B |  | 2014_I | 1.68 | A |
|  | 2015_NI | 1.02 | C |  | 2015_I | 0.44 | B |
|  | 2015_I | 1 | C |  | 2015_NI | 0.43 | B |
| FA-epigallocat | 2014_I | 1.2 | A |  | 2014_NI | 0.64 | A |
|  | 2014_NI | 0.99 | A B |  | 2014_I | 0.57 | A |
|  | 2015_I | 0.87 | A B |  | 2015_I | 0.42 | A B |
|  | 2015_NI | 0.8 | B |  | 2015_NI | 0.36 | B |
| FA-epicat | 2015_I | 4.49 | A |  | 2015_I | 1.99 | A |
|  | 2014_I | 4.07 | A |  | 2014_I | 1.7 | A |
|  | 2015_NI | 3.88 | A |  | 2015_NI | 1.6 | A |
|  | 2014_NI | 2.73 | B |  | 2014_NI | 1.59 | A |
| FA-cat | 2015_I | 25.1 | A |  | 2015_I | 10.72 | A |
|  | 2015_NI | 21.63 | A |  | 2015_NI | 8.53 | B |
|  | 2014_I | 15.61 | B |  | 2014_I | 6.27 | C |
|  | 2014_NI | 10.68 | C |  | 2014_NI | 5.86 | C |
| FA-(epi)cat-eth-(epi)cat-i1 | 2015_I | 0.15 | A |  | 2015_I | 0.06 | A |
|  | 2015_NI | 0.14 | A |  | 2015_NI | 0.06 | A |
|  | 2014_I | 0.05 | B |  | 2014_NI | 0.02 | B |
|  | 2014_NI | 0.04 | B |  | 2014_I | 0.02 | B |
| FA-(epi)cat-eth-(epi)cat-i2+3 | 2015_NI | 0.27 | A |  | 2015_NI | 0.12 | A |
|  | 2015_I | 0.22 | A B |  | 2015_I | 0.09 | A |
|  | 2014_I | 0.13 | B |  | 2014_NI | 0.08 | A |
|  | 2014_NI | 0.11 | B |  | 2014_I | 0.06 | A |
| FA-gallocat-term | 2014_I | 30.77 | A |  | 2014_NI | 16.14 | A |
|  | 2015_NI | 29.31 | A |  | 2014_I | 12.92 | B |
|  | 2014_NI | 27.99 | A |  | 2015_NI | 11.8 | B |
|  | 2015_I | 26.62 | A |  | 2015_I | 11.49 | B |
| FA-epigallocat-term | 2014_I | 5.51 | A |  | 2014_NI | 3.16 | A |
|  | 2014_NI | 5.27 | A |  | 2014_I | 2.4 | B |
|  | 2015_NI | 4.85 | A |  | 2015_NI | 2.03 | B |
|  | 2015_I | 4.53 | A |  | 2015_I | 2.01 | B |
| FA-epicat-term | 2015_I | 13.85 | A |  | 2015_I | 5.91 | A |
|  | 2015_NI | 12.94 | A |  | 2015_NI | 5.26 | A B |
|  | 2014_I | 10.2 | B |  | 2014_NI | 4.57 | B |
|  | 2014_NI | 7.91 | C |  | 2014_I | 4.18 | B |
| FA-epicat-gall-term | 2015_NI | 5.76 | A |  | 2015_NI | 2.31 | A |
|  | 2015_I | 5.53 | A |  | 2015_I | 2.27 | A |
|  | 2014_I | 3.64 | B |  | 2014_NI | 2 | A B |
|  | 2014_NI | 3.41 | B |  | 2014_I | 1.53 | B |
| FA-cat-term | 2015_I | 193.52 | A |  | 2015_I | 80.29 | A |
|  | 2015_NI | 179.95 | A |  | 2015_NI | 71.52 | A |
|  | 2014_I | 113.72 | B |  | 2014_NI | 45.81 | B |
|  | 2014_NI | 81.84 | C |  | 2014_I | 45.7 | B |
| FA-(epi)gallocat-phlo | 2015_NI | 1089.03 | A |  | 2014_NI | 531.3 | A |
|  | 2015_I | 1000.08 | A B |  | 2015_NI | 465.18 | A B |
|  | 2014_I | 906.23 | B |  | 2015_I | 448.16 | B |
|  | 2014_NI | 885.84 | B |  | 2014_I | 392.26 | B |
| FA-epicat-phlo | 2015_I | 2428.05 | A |  | 2015_I | 1051.44 | A |
|  | 2015_NI | 2337.29 | A |  | 2015_NI | 980.72 | A |
|  | 2014_I | 1897.55 | B |  | 2014_NI | 913.46 | A |
|  | 2014_NI | 1586.4 | C |  | 2014_I | 786.11 | A B |
| FA-epicat-gall-phlo | 2015_I | 109.72 | A |  | 2014_NI | 55.72 | A |
|  | 2015_NI | 107.83 | A |  | 2015_I | 48.05 | A B |
|  | 2014_I | 104.2 | A |  | 2015_NI | 45.21 | B |
|  | 2014_NI | 94.41 | A |  | 2014_I | 44.39 | B |
| FA-cat-phlo | 2015_I | 16.18 | A |  | 2015_I | 6.91 | A |
|  | 2015_NI | 15.64 | A |  | 2015_NI | 6.4 | A |
|  | 2014_I | 9.2 | B |  | 2014_NI | 4.45 | B |
|  | 2014_NI | 7.69 | B |  | 2014_I | 3.8 | B |
| HB-glucogall | 2015_NI | 1.83 | A |  | 2015_NI | 0.73 | A |
|  | 2015_I | 1.53 | A |  | 2015_I | 0.61 | B |
|  | 2014_I | 0.65 | B |  | 2014_NI | 0.36 | C |
|  | 2014_NI | 0.63 | B |  | 2014_I | 0.26 | C |
| HB-vanill-ac | 2015_I | 0.27 | A |  | 2015_I | 0.11 | A |
|  | 2015_NI | 0.21 | A |  | 2015_NI | 0.09 | A B |
|  | 2014_I | 0.1 | B |  | 2014_NI | 0.06 | B C |
|  | 2014_NI | 0.09 | B |  | 2014_I | 0.04 | C |
| HB-syring-ac | 2015_NI | 0.8 | A |  | 2015_NI | 0.38 | A |
|  | 2015_I | 0.74 | A |  | 2015_I | 0.34 | A |
|  | 2014_I | 0.33 | B |  | 2014_NI | 0.23 | A B |
|  | 2014_NI | 0.31 | B |  | 2014_I | 0.15 | B |
| HB-protocat-ac | 2015_I | 0.49 | A |  | 2015_I | 0.2 | A |
|  | 2015_NI | 0.42 | A |  | 2015_NI | 0.16 | A |
|  | 2014_I | 0.14 | B |  | 2014_NI | 0.08 | B |
|  | 2014_NI | 0.14 | B |  | 2014_I | 0.06 | B |
| HB-gall-ac | 2015_NI | 0.73 | A |  | 2015_NI | 0.31 | A |
|  | 2015_I | 0.72 | A |  | 2015_I | 0.3 | A |
|  | 2014_NI | 0.26 | B |  | 2014_NI | 0.16 | B |
|  | 2014_I | 0.26 | B |  | 2014_I | 0.11 | B |
| HC-ct-coutar-ac | 2015_I | 110.44 | A |  | 2015_I | 46.73 | A |
|  | 2015_NI | 107.01 | A |  | 2015_NI | 44.47 | A |
|  | 2014_I | 85.79 | B |  | 2014_NI | 42.72 | A |
|  | 2014_NI | 74.22 | B |  | 2014_I | 35.63 | A |
| HC-ct-caftar-ac | 2015_I | 229.87 | A |  | 2015_I | 96.99 | A |
|  | 2015_NI | 223.43 | A |  | 2015_NI | 93.45 | A |
|  | 2014_I | 139.62 | B |  | 2014_NI | 68.97 | B |
|  | 2014_NI | 116.68 | B |  | 2014_I | 58.94 | B |
| HC-t-fertar-ac | 2015_NI | 10.57 | A |  | 2015_NI | 4.24 | A |
|  | 2015_I | 10.3 | A |  | 2015_I | 4.17 | A |
|  | 2014_NI | 4.41 | B |  | 2014_NI | 2.43 | B |
|  | 2014_I | 4.32 | B |  | 2014_I | 1.71 | B |
| HC-t-caffeic-ac | 2015_NI | 0.15 | A |  | 2015_NI | 0.06 | A |
|  | 2015_I | 0.15 | A |  | 2015_I | 0.06 | A |
|  | 2014_I | 0.04 | B |  | 2014_NI | 0.02 | B |
|  | 2014_NI | 0.03 | B |  | 2014_I | 0.02 | B |
| HC-t-coumar-ac | 2015_I | 0.19 | A |  | 2015_I | 0.08 | A |
|  | 2015_NI | 0.18 | A |  | 2015_NI | 0.07 | A |
|  | 2014_I | 0.06 | B |  | 2014_NI | 0.03 | B |
|  | 2014_NI | 0.06 | B |  | 2014_I | 0.02 | B |
| HC-t-ferul-ac | 2015_I | 0.17 | A |  | 2015_I | 0.06 | A |
|  | 2015_NI | 0.15 | A |  | 2015_NI | 0.06 | A |
|  | 2014_I | 0.04 | B |  | 2014_NI | 0.02 | B |
|  | 2014_NI | 0.03 | B |  | 2014_I | 0.01 | B |
| OT-OH-tyrosol | 2015_I | 0.16 | A |  | 2015_I | 0.06 | A |
|  | 2015_NI | 0.15 | A |  | 2015_NI | 0.06 | A |
|  | 2014_I | 0.07 | B |  | 2014_NI | 0.03 | B |
|  | 2014_NI | 0.06 | B |  | 2014_I | 0.03 | B |
| OT-GSSG | 2015_I | 3.2 | A |  | 2015_I | 1.33 | A |
|  | 2015_NI | 3.08 | A |  | 2015_NI | 1.3 | A |
|  | 2014_I | 2.18 | B |  | 2014_I | 0.95 | B |
|  | 2014_NI | 1.66 | C |  | 2014_NI | 0.94 | B |
| OT-GSH | 2015_I | 10,27 | A |  | 2015_I | 4.59 | A |
|  | 2015_NI | 9.05 | A |  | 2015_NI | 3.84 | A |
|  | 2014_I | 5.24 | B |  | 2014_I | 2.69 | B |
|  | 2014_NI | 4.52 | B |  | 2014_NI | 2.36 | B |
|  |  |  |  |  |  |  |  |
|  | | | | |  |  |  |
| s_AN_n | 2015_NI | 937.05 | A |  | 2014_NI | 502.66 | A |
|  | 2015_I | 837.25 | A |  | 2015_NI | 439.8 | A |
|  | 2014_I | 734.97 | A |  | 2015_I | 398.35 | A |
|  | 2014_NI | 730.63 | A |  | 2014_I | 357.84 | A |
| s_FA | 2015_I | 3798.1 | A |  | 2015_I | 1656.57 | A |
|  | 2015_NI | 3782.64 | A |  | 2015_NI | 1590.45 | A |
|  | 2014_I | 3081.06 | B |  | 2014_NI | 1576.64 | A |
|  | 2014_NI | 2700.71 | B |  | 2014_I | 1293.33 | B |
| s_FV | 2015_NI | 707.03 | A |  | 2014_NI | 289.51 | A |
|  | 2015_I | 677.65 | A |  | 2015_I | 264.05 | A B |
|  | 2014_I | 527.32 | B |  | 2015_NI | 263.79 | A B |
|  | 2014_NI | 508.17 | B |  | 2014_I | 215.36 | B |
| s_HB | 2015_NI | 4.01 | A |  | 2015_NI | 1.69 | A |
|  | 2015_I | 3.77 | A |  | 2015_I | 1.58 | A |
|  | 2014_I | 1.49 | B |  | 2014_NI | 0.91 | B |
|  | 2014_NI | 1.44 | B |  | 2014_I | 0.64 | B |
| s_HC | 2015_I | 351.14 | A |  | 2015_I | 148.12 | A |
|  | 2015_NI | 341.51 | A |  | 2015_NI | 142.38 | A |
|  | 2014_I | 229.9 | B |  | 2014_NI | 114.21 | B |
|  | 2014_NI | 195.45 | B |  | 2014_I | 96.36 | B |
| s_ST | 2015_I | 44.13 | A |  | 2014_NI | 22.99 | A |
|  | 2015_NI | 42.76 | A |  | 2015_I | 18.71 | A |
|  | 2014_I | 38.88 | A |  | 2015_NI | 18.19 | A |
|  | 2014_NI | 37.19 | A |  | 2014_I | 15.47 | A |

| **Qualitative polyphenolic indices** | |  |  |  |  |  |  |
| --- | --- | --- | --- | --- | --- | --- | --- |
| p_AN_acyl (%) | 2015_NI | 47.9 | A |  |  |  |  |
|  | 2015_I | 46.86 | A |  |  |  |  |
|  | 2014_I | 42.33 | B |  |  |  |  |
|  | 2014_NI | 38.66 | B |  |  |  |  |
| p_AN_tri (%) | 2014_I | 57.72 | A |  |  |  |  |
|  | 2014_NI | 57.54 | A |  |  |  |  |
|  | 2015_NI | 56.9 | A |  |  |  |  |
|  | 2015_I | 54.92 | A |  |  |  |  |
| p_AN_met (%) | 2015_NI | 58.24 | A |  |  |  |  |
|  | 2014_I | 57.57 | A |  |  |  |  |
|  | 2015_I | 57.18 | A |  |  |  |  |
|  | 2014_NI | 56.49 | A |  |  |  |  |
| p_FA_gall (%) | 2014_NI | 3.6 | A |  |  |  |  |
|  | 2014_I | 3.46 | A |  |  |  |  |
|  | 2015_I | 3.19 | A |  |  |  |  |
|  | 2015_NI | 3.15 | A |  |  |  |  |
| p_FA_tri (%) | 2014_NI | 34.65 | A |  |  |  |  |
|  | 2014_I | 30.96 | B |  |  |  |  |
|  | 2015_NI | 28.64 | C |  |  |  |  |
|  | 2015_I | 25.72 | D |  |  |  |  |
| p_FV_mono (%) | 2014_NI | 29.38 | A |  |  |  |  |
|  | 2014_I | 28.44 | A |  |  |  |  |
|  | 2015_NI | 27.25 | A |  |  |  |  |
|  | 2015_I | 26.09 | A |  |  |  |  |
| p_FV_di (%) | 2015_I | 67.46 | A |  |  |  |  |
|  | 2015_NI | 65.49 | A B |  |  |  |  |
|  | 2014_I | 63.78 | B |  |  |  |  |
|  | 2014_NI | 63.15 | B |  |  |  |  |
| p_FV_tri (%) | 2014_I | 7.76 | A |  |  |  |  |
|  | 2014_NI | 7.45 | A |  |  |  |  |
|  | 2015_NI | 7.24 | A |  |  |  |  |
|  | 2015_I | 6.43 | A |  |  |  |  |
| p_FV_met (%) | 2014_I | 7.81 | A |  |  |  |  |
|  | 2014_NI | 7.79 | A |  |  |  |  |
|  | 2015_NI | 6.12 | A |  |  |  |  |
|  | 2015_I | 5.22 | A |  |  |  |  |
| p_FV_glucur (%) | 2015_I | 19.57 | A |  |  |  |  |
|  | 2015_NI | 18.09 | A |  |  |  |  |
|  | 2014_I | 18.08 | A |  |  |  |  |
|  | 2014_NI | 17.04 | A |  |  |  |  |
| dp_FA | 2014_NI | 23.62 | A |  |  |  |  |
|  | 2014_I | 21.33 | B |  |  |  |  |
|  | 2015_NI | 19.18 | C |  |  |  |  |
|  | 2015_I | 18.53 | C |  |  |  |  |
| **Other parameters** |  |  |  |  |  |  |  |
| deltaC13 | 2014_NI | -23.67 | A |  |  |  |  |
|  | 2014_I | -25.21 | B |  |  |  |  |
|  | 2015_NI | -25.5 | B |  |  |  |  |
|  | 2015_I | -26.94 | C |  |  |  |  |
|  |  |  |  |  |  |  |  |
| Sugar content (°Brix) | 2014_NI | 20.17 | A |  |  |  |  |
|  | 2014_I | 19.74 | A B |  |  |  |  |
|  | 2015_I | 19.3 | B |  |  |  |  |
|  | 2015_NI | 19.22 | B |  |  |  |  |
|  |  |  |  |  |  |  |  |
| Berry weight (g) | 2015_I | 2.76 | A |  |  |  |  |
|  | 2015_NI | 2.66 | A |  |  |  |  |
|  | 2014_I | 2.59 | A |  |  |  |  |
|  | 2014_NI | 1.87 | B |  |  |  |  |
| * groups : |  |  |  |  |  |  |  |
| 2014_NI : year 2014, not irrigated, 107 observations | | | | |  |  |  |
| 2014_I : year 2014, irrigated, 107 observations | | | |  |  |  |  |
| 2015_NI : year 2015, not irrigated, 107 observations | | | |  |  |  |  |
| 2015_I : year 2015, irrigated, 107 observations | | | |  |  |  |  |
|  | | | | | | | |
