# Supplementary material for: Cultivar Diversity of Grape Skin Polyphenol Composition and Changes in Response to Drought Investigated by LC-MS Based Metabolomics
Source: Front Plant Sci. 2017 Oct 27;8:1826. doi: 10.3389/fpls.2017.01826 (PMC5663694; doi:10.3389/fpls.2017.01826)
Supplement: Supplementary file 3 [file Table3.DOCX]

Supplementary Material

Cultivar diversity of grape skin polyphenol composition and changes in response to drought investigated by LC-MS based metabolomics

Lucie Pinasseau, Anna Vallverdú-Queralt, Arnaud Verbaere, Maryline Roque, Emmanuelle Meudec, Loïc Le Cunff, Jean-Pierre Péros, Agnès Ageorges, Nicolas Sommerer, Jean-Claude Boulet, Nancy Terrier, Véronique Cheynier^*^

*** Correspondence:** Corresponding Author: [veronique.cheynier@inra.fr](mailto:veronique.cheynier@inra.fr)

**Supplementary Table S3**: sample distribution in the 8 cultivar subgroups arising from non-hierarchical clustering of metabolites and cultivars affected by drought, calculated for all variables (Log (content I/content NI), with polyphenol concentrations expressed in mg berry^-1^) on the 2014 data set (Figure 7) and p-values of Chi-square tests performed on each subgroup and on the entire population for color (colored, white), genetic group (wine West, wine East, table East), and harvest dates under not irrigated (NI) and irrigated (I) conditions (before Aug 31^st^, Sept 1^st^ through Sept 20^th^, after Sept 20^th^). Bold characters indicate p-values below 0.05.

|  | number of cultivars | Chi-square color | Chi-square genetic group | Chi-square harvest date (NI) | Chi-square harvest date (I) |
| --- | --- | --- | --- | --- | --- |
| Group 1-1-1 | 16 | 0.409 | 0.92 | 0.57 | 0.99 |
| Group 1-1-2 | 34 | 0.186 | 0.33 | 0.07 | 0.63 |
| Group 1-2-1 | 22 | **0.003** | 0.69 | 0.06 | 0.06 |
| Group 1-2-2 | 28 | **0.043** | 0.41 | 0.73 | 0.57 |
| Group 2-1-1 | 27 | **0.005** | 0.47 | 0.06 | 0.21 |
| Group 2-1-2 | 32 | 0.634 | **0.02** | 0.91 | 0.61 |
| Group 2-2-1 | 17 | 0.534 | 0.13 | **0.04** | 0.69 |
| Group 2-2-2 | 30 | **9.08496E-05** | 0.78 | 0.08 | 0.18 |
| Whole population | 206 | **1.68305E-06** | 0.17 | **0.01** | 0.32 |
